# Longitudinal changes in reinforcement learning during smoking cessation: a computational analysis using a probabilistic reward task

**DOI:** 10.1038/s41598-024-84091-y

**Published:** 2024-12-31

**Authors:** Chiara Montemitro, Paolo Ossola, Thomas J. Ross, Quentin J. M. Huys, John R. Fedota, Betty Jo Salmeron, Massimo di Giannantonio, Elliot A. Stein

**Affiliations:** 1 Neuroimaging Research Branch, National Institute on Drug Abuse-Intramural Research Program, National Institutes of Health, Baltimore, MD USA; 2 Department of Neuroscience, Imaging and Clinical Sciences, “G. d’Annunzio” University, Chieti, Italy; 3 Present Address: Center for Social and Affective Neuroscience, Linköping University, Linköping, Sweden; 4 Department of Medicine and Surgery, University of Parma, Parma, Italy; 5 Department of Mental Health, AUSL Parma, Parma, Italy; 6 Applied Computational Psychiatry Laboratory, Mental Health Neuroscience Department, Division of Psychiatry and Max Planck Centre for Computational Psychiatry and Ageing Research, Queen Square Institute of Neurology, University College London, London, UK; 7 Present Address: Behavioral and Cognitive Neuroscience Branch, Division of Neuroscience and Behavior, National Institute on Drug Abuse, National Institutes of Health, Bethesda, MD USA

**Keywords:** Smoking cessation, Reinforcement learning, Decision-making, Withdrawal, Nicotine abstinence, Punishment sensitivity, Decision, Outcomes research, Addiction

## Abstract

Despite progress in smoking reduction in the past several decades, cigarette smoking remains a significant public health concern world-wide, with many smokers attempting but ultimately failing to maintain abstinence. However, little is known about how decision-making evolves in quitting smokers. Based on preregistered hypotheses and analysis plan (https://osf.io/yq5th), we examined the evolution of reinforcement learning (RL), a key component of decision-making, in smokers during acute and extended nicotine abstinence. In a longitudinal, within-subject design, we used a probabilistic reward task (PRT) to assess RL in twenty smokers who successfully refrained from smoking for at least 30 days. We evaluated changes in reward-based decision-making using signal-detection analysis and five RL models across three sessions during 30 days of nicotine abstinence. Contrary to our preregistered hypothesis, punishment sensitivity emerged as the only parameter that changed during smoking cessation. While it is plausible that some changes in task performance could be attributed to task repetition effects, we observed a clear impact of the Nicotine Withdrawal Syndrome (NWS) on RL, and a dynamic relationship between craving and reward and punishment sensitivity over time, suggesting a significant recalibration of cognitive processes during abstinence. In this context, the heightened sensitivity to negative outcomes observed at the last session (30 days after quitting) compared to the previous sessions, may be interpreted as a cognitive adaptation aimed at fostering long-term abstinence. While further studies are needed to clarify the mechanisms underlying punishment sensitivity during nicotine abstinence, these results highlight the need for personalized treatment approaches tailored to individual needs.

**Supplementary Information:**

The online version contains supplementary material available at 10.1038/s41598-024-84091-y.

## Introduction

The majority of cigarette smokers express a desire to quit smoking: approximately 78% indicate an interest in quitting^1^, and over 40% of smokers have made at least one quit attempt within the past year^1^. Despite these efforts, the success rate remains low, as around 65% of individuals who attempt to quit ultimately relapse within 30 days^1,2^. A significant contributor to this challenge is the Nicotine Withdrawal Syndrome (NWS), a constellation of symptoms that encompass heightened negative affect, irritability, lapses of attention, and cravings^2,3^. These symptoms manifest soon after smoking cessation and peak within the first week^2,3^, gradually diminishing as abstinence progresses^4^. Nevertheless, some smokers continue to relapse even after an extended period of abstinence^2^. Since the pursuit of sustained abstinence demands smokers to make decisions aligned with their long-term goals, despite the considerable challenges posed by NWS, understanding how decision-making evolves in quitting smokers is of paramount importance.

Reinforcement learning (RL)^5^, an essential component of decision-making^6,7^, plays a crucial role in maintaining abstinence. At its core, RL involves the continuous evaluation of outcomes and the processing of feedback^8^, and the recalibration of the decision-making mechanisms to shape future choices based on past experiences and long-term goals^8^. Nicotine, like other stimulant drugs, alters this process^7^, increasing reward sensitivity and responsiveness^9^, and acting both as a primary reinforcer and a salience-enhancer of other rewards^10^. Conversely, reward responsiveness is usually reduced during nicotine withdrawal^11–13^, when learning from negative prediction error signals seems increased^13^. However, longitudinal studies evaluating RL during smoking cessation protocols are lacking, and little is known about how RL evolves in quitting smokers and how individuals adapt their decision-making strategies accordingly.

To address these knowledge gaps, we collected behavioral data from individuals performing a probabilistic reward task (PRT;^14^) as part of an extensive and ongoing smoking cessation protocol (NCT01867411). Longitudinal changes in PRT performance have been previously reported dysregulated following acute nicotine abstinence^12,15,16^ but never evaluated following more extended abstinence. Further, PRT offers the opportunity to apply RL models allowing to evaluate individual’s ability to learn and adapt behavior based on feedback and reward probability^14^.

Our main aim was to evaluate changes in reward sensitivity and learning rate throughout the course of nicotine withdrawal and identify the relationship between reward-related behavior and measures of withdrawal. We hypothesized the propensity to modulate behavior as a function of prior reinforcement to be affected by both acute (48 h) and extended (30 days) nicotine abstinence. Specifically, we expected both acute and extended abstinence to decrease sensitivity to rewards compared to the satiated state. To increase the reliability of data analysis^17^, hypotheses and methods were pre-registered before calculating any descriptive statistics or conducting inferential tests. These are available in full at https://osf.io/yq5th.

## Methods

### Experimental design

In a longitudinal, within-subjects design, treatment-seeking smokers were enrolled in a smoking cessation protocol evaluating possible biomarkers of nicotine dependence severity that may be useful in predicting success in smoking cessation (ClinicalTrials.gov NCT01867411 registered on 06/04/2013). All methods were performed in accordance with the relevant guidelines and regulations, and approved by the National Institute on Drug Abuse, Intramural Research Program Institutional Review Board. Written informed consent was obtained in accordance with the National Institute on Drug Abuse, Intramural Research Program Institutional Review Board.

As part of this larger, ongoing, smoking cessation protocol, twenty (14 male) treatment-seeking smokers were able to successfully quit smoking and remain abstinent for at least 30 days, and were evaluated at study entry (S0), at peak (48 h) withdrawal (ABS2), and at one month into their treatment-supported, biochemically-verified extended abstinence (ABS30).

Smokers were instructed to smoke ad lib before S0 (last cigarette smoked on average 50 (5–187) minutes before session), to abstain from smoking two days before ABS2 (49.5 ± 1.5 h), and to remain abstinent 30 days before the ABS30 session. Smoking status was biochemically verified at each session by carbon monoxide (CO) breath testing (BreathCO, Vitalograph, Lenexa, KS) (S0: 20.2 ± 12.4; ABS2: 2.1 ± 0.5; ABS30: 3.1 ± 3.5 ppm).

After ABS2, smokers returned to smoking as usual behavior and, to improve the likelihood of prolonged abstinence, were offered counselling based on motivational interviewing and cognitive behavioral techniques, which generally continued weekly from ABS2 for the next 12 weeks. During these interviews, subjects set a quit day (from 2 to 4 weeks after ABS2) together with their coach and were offered e-cigarettes (primarily) or nicotine replacement (NRT) for 12 days before the quitting day, to aid in their transition to abstinence. Those subjects who accepted e-cigarette smoking were tested once more at the end of the 12-day period, but this was not mandatory and not all subjects included in the current analyses (16 out of 20) were tested at this timepoint. The last session was scheduled 30 days after the quit day. Note, the effectiveness of these interventions is not of interest in this analysis; they were provided to improve the likelihood of sustained abstinence which was necessary to test our RL hypotheses.

**Fig. 1:**
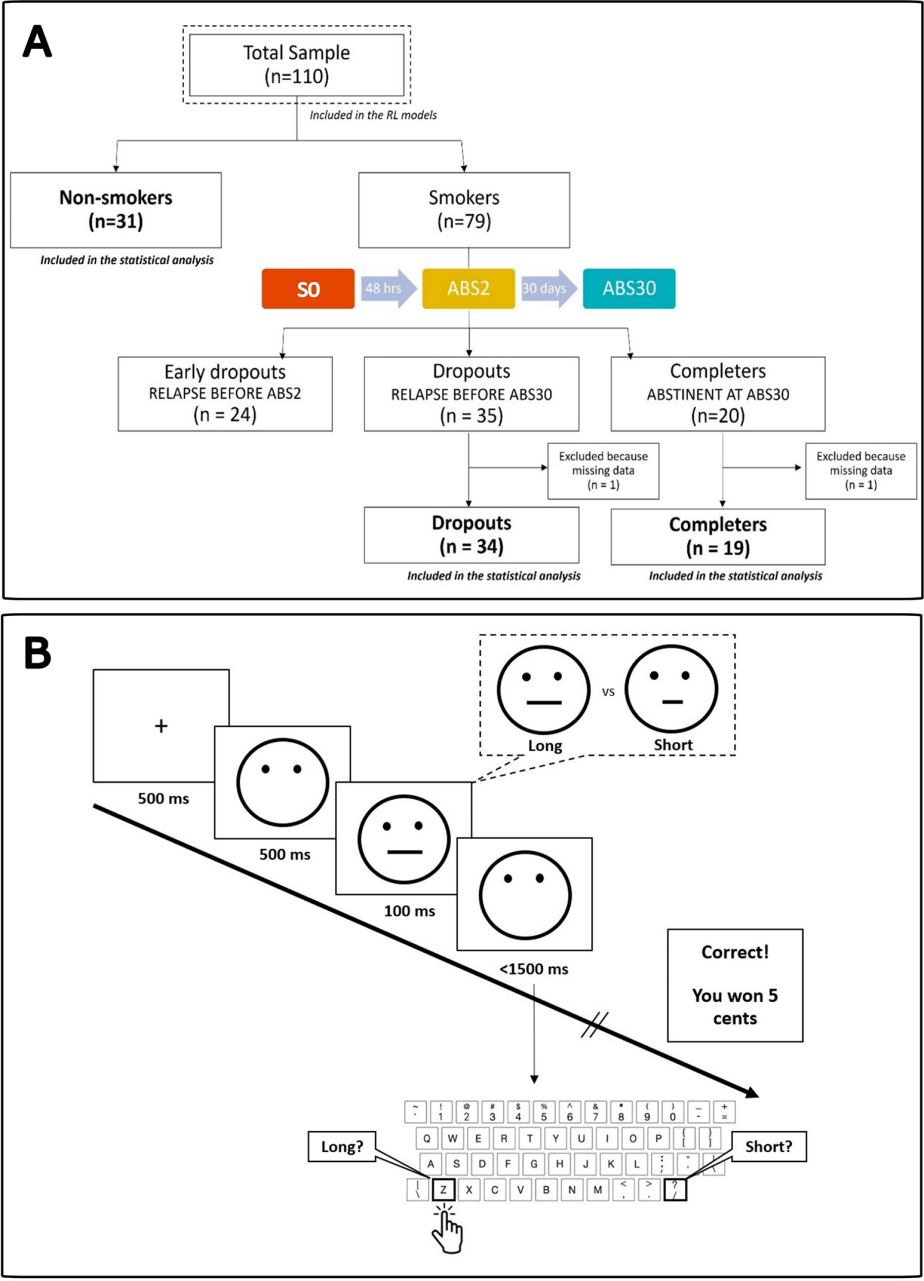
Study methods. (**A**) Study flowchart: task behavioral data from all participants (*n* = 110) were included in RL computational model fitting procedures. Statistical analysis was performed on 84 subjects: 19 completers who performed the task three times (S0, ABS2, and ABS30), 34 dropouts who performed the task twice (S1 and ABS2) and 31 healthy controls who performed the task once. (**B**) Probabilistic Reward Learning task structure.

Although we preregistered five hypotheses, this manuscript focuses on testing and reporting on only one of them: the changes in punishment and reward sensitivity in those subjects who successfully refrained from smoking for 30 days and underwent evaluation at ABS30, regardless to their participation in the e-cigarettes protocol (*n* = 19). The remaining hypotheses have been assessed separately, with results to be reported in additional publications to follow. Under the same protocol, data were also collected from smokers who relapsed right after acute abstinence (S0 and ABS2, *n* = 35), and early dropout smokers who performed the task only once (S0, *n* = 24). Matched non-smoking, healthy participants were evaluated only once (S0, *n* = 31) (see Fig. 1A). Analyses of these control participants and dropouts were not defined in the pre-registration and have been added only to evaluate the directionality of changes observed during smoking cessation and test whether such changes in reward processing ‘return’ to levels seen in non-smokers.

### Study procedures

Prior to study initiation, all participants underwent medical and psychometric assessment (see Supplementary Methods). Nicotine addiction severity and motivation to quit were measured with the Fagerstrom Test for Nicotine Dependence (FTND)^18^ and the Decision Balance Worksheet for Smoking (DBW), respectively^19^.

At the beginning of each session, participants underwent a brief clinical evaluation, including breathalyzer, CO test, urine tests for common drugs of abuse, a metal/MRI safety screening, and if female, a urine pregnancy test. Participants also reported their subjective perception of withdrawal symptoms and tobacco craving through validated instruments (Wisconsin Smoking Withdrawal Scale [WSWS^20^, and Tobacco Craving Questionnaire [TCQ^21^, respectively).

During each session, participants spent about three hours in the MRI scanner where several tasks were administered. Details of the scanning and task procedures are out of the scope of the current manuscript and are fully available at https://osf.io/yq5th. At the end of each session, after completing all scanning procedures, participants performed, among other tasks, a Probabilistic Reward Task (PRT)^14^, used to probe RL.

### Probabilistic reward task

Briefly, the PRT consisted of three blocks of 100 trials eachwith a simple line-drawing face presented with either a short (11.5 mm) or long (13 mm) mouth for 100 ms. Participants were required to indicate the mouth length and received positive feedback for only 40% of correct responses (“Correct!! You won 20pts”) (Fig. 1B) with an asymmetrical reinforcement schedule (correct responses to one stimulus were rewarded three times more than correct responses to the other). This asymmetry in the reinforcement schedule is responsible for systematic preference towards the most rewarded stimulus, namely, *response bias*. Importantly, subjects had no information about the asymmetry, they were only instructed to win as much as possible. Task stimuli were presented using E-Prime software (Psychology Software Tools, Sharpsburg, PA).

To compare our sample to previous studies, outside of the preregistered analysis plan, we first applied model-free signal-detection analysis methods^14,22^. Based on the randomization in the pairing between the stimuli (short vs. long) and the probability of getting a reward, the stimuli were classified as *rich* and *lean*, and six behavioral variables of interest were calculated: *Reward Bias* (RB), indicating responsiveness to rewards and the subject’s tendency to report the rich compared to the lean stimuli; *Discriminability Rate* (DR), indicating individual’s ability to discriminate between the two stimuli; *Cumulative Reward (CR)* representing the total rewards accumulated by the subject, alongside *Accuracy for Rich* (AR) and *Lean* (AL) *stimuli* ;and the overall participants’ accuracy expressed as the percentage of correct responses (*Fraction Correct Responses* (FC)). For each subject we also computed the mean *Reaction Time (RT)* for rich and lean stimuli, based on response accuracy. Details about model free variables are provided in Supplementary Methods.

Additionally, five classic reinforcement learning models^23^ were fit to the behavioral data from 110 subjects enrolled in the study. Based on the specific role of negative reinforcement in nicotine addiction and withdrawal^24^, our initial plan was to evaluate only two (*Punishment* and *Belief*) of the five models. However, to enhance methodological rigor and minimize the risk of misinterpretation, we decided to fit and compare all five RL models to our data. As previously reported^25^, choices were modelled with a softmax decision function that expresses selection probabilities as a sigmoidal function. The five models (*Action-only*, *Stimulus-action*, *Punishment*, *Belief*, *and Counterfactual*)^25^ included different combinations of six behavioral parameters: reward learning rate (α), initial action bias (Q_0_), two reward sensitivities (β_reward_, β_punishment_), instruction sensitivity (γ), and a belief parameter (ζ). Model parameters, model-fitting procedures, and models’ comparison on their ability to explain the data are detailed in the Supplementary Methods.

### Statistical analyses

Two software programs, R (version: 3.6.3, R-project.org) and Matlab (version: 9.13.0 (R2022b), The MathWorks Inc.; 2022), were used for model fitting and statistical analyses. From the original sample (*n* = 110), statistical analyses aimed at testing the preregistered hypotheses were performed on 19 completers who successfully achieve 30-day abstinence and performed the task three times (S0, ABS2, and ABS30) (see Fig. 1). Non-smoking healthy controls (*n* = 31) and smokers who dropped-out before ABS30 (*n* = 59) were included in models fitting procedures and in supplementary analyses to explore the directionality of observed effects beyond the preregistered hypotheses (see Supplementary Methods and Results).

To ascertaining the effects of abstinence on a subjective level we first employed repeated-measures ANOVAs for subjective withdrawal measures (WSWS) and craving (TCQ) within completers, using sessions (S0, ABS2, ABS30) as a within-subject factor. We then investigated PRT performance indices changes over time. Specifically, repeated-measures ANOVAs were used to test the effects of sessions on the six model-free variables. Further, a three-way ANOVA with sessions, conditions (rich or lean) and accuracy as within-subject factors was used to evaluate the changes in RT across sessions.

Repeated-measures ANOVAs were also used to investigate changes in the winning model’s parameters within completers, using sessions as a within-subject factor. The parameters of interest were entered into the analyses as log-transformed variables: log(α/(1-α)), log(Q_0_), log(β_reward_), log(β_punishment_), log(γ). Statistically significant effects were controlled by including other model parameters as covariates.

Beyond the a-priori hypotheses, we were interested in comparing completers and dropouts. Two-by-two repeated-measures ANOVAs with sessions (S0, ABS2) as within-subject factor and group (completers, dropouts) as between-subject factor were used to compare the effects of acute abstinence on WSWS, TCQ, and PRT performance between completers and dropouts. Also, to determine the directionality of the observed effects, we compared completers and dropouts at baseline with a group of matched non-smoking controls who underwent testing only once. One-way ANOVAs with ‘group’ (completers, dropouts, controls) as the between-subject factor were used for this comparison.

All post-hoc analyses were performed using Student’s t-tests, and Hommel-corrected significance threshold of *p* < 0.05 was applied to all tests.

To evaluate the effects of addiction severity (FTND) and motivation to quit (DBW-S) on RL, multiple linear robust regressions between the demeaned values of the questionnaires administered at baseline and the change in model’s parameters during acute (ABS2 vs. S0) and extended (ABS30 vs. S0) nicotine abstinence were performed. Last, we tested the association between model’s parameters and craving and NWS, by conducting a set of Pearson’s correlations at each timepoint and within-subject correlations across multiple timepoints using repeated-measure correlations.

## Results

### Withdrawal symptoms and nicotine craving fluctuates across sessions, in line with nicotine abstinence

**Fig. 2:**
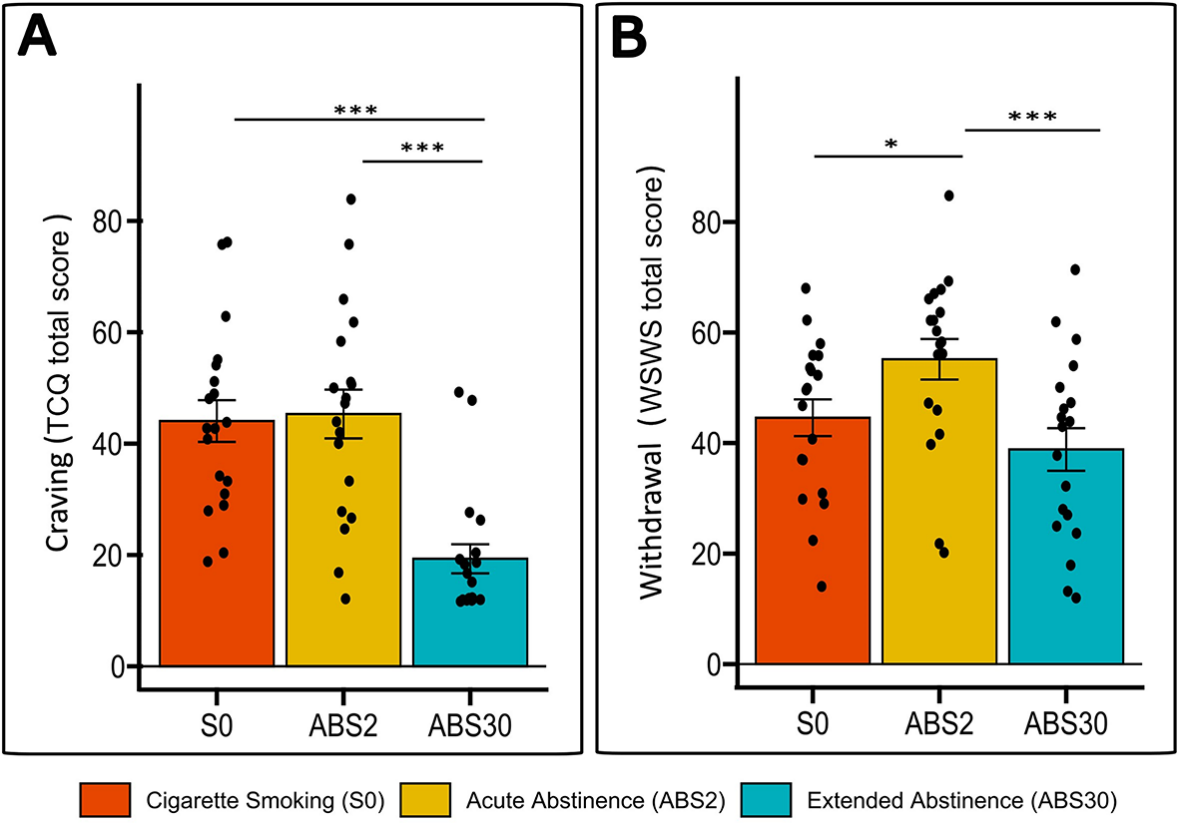
Abstinence-induced changes in measures of craving and withdrawal. Mean self-report measures of nicotine craving (TCQ) (**A**) and withdrawal symptoms (WSWS) (**B**) in completers by smoking status. Error bars represent standard errors. Black bars identify the significant differences (*p < 0.05, ** *p* < 0.01, *** *P* < 0.001) at pairwise comparison when the ANOVA was significant.

All smokers, both completers and dropouts, were severely addicted to nicotine, with a long smoking history and a large consumption of cigarettes per day (see Table 1). They did not show symptoms of depression at baseline, although five subjects (2 completers, 2 dropouts and 1 non-smoking control) reported at least one previous episode of depression. In line with the pre-registered hypotheses and the extant literature^3,26^, subjective ratings of withdrawal were modulated by nicotine abstinence in a time-dependent fashion (Fig. 2). Among completers, abstinence altered self-reported craving (TCQ; F = 26.26, *p* < 0.001), with TCQ being markedly lower at extended abstinence (S0 vs. ABS30: *p* < 0.001, ABS2 vs. ABS30: *p* < 0.001), although it did not differ between satiety (S0) and acute abstinence (ABS2) (Fig. 2A). While no overall effect of time on withdrawal symptoms (WSWS) was observed (F = 2.5, *p* = 0.12), WSWS increased following acute abstinence (S0 vs. ABS2: *p* = 0.012) and decreased following ABS30 (S0 vs. ABS30: *p* = 0.084, ABS2 vs. ABS30: *p* < 0.001), such that withdrawal symptoms at ABS30 no longer differed from satiety (Fig. 2B).

Notably when comparing between completers and dropouts, we observed a Session × Group interaction on TCQ (F = 5.051, *p* = 0.027), such that dropouts, but not completers, experienced increased craving between S0 and ABS2 (*p* < 0.001). Although limited by the small sample size, these results suggest that subjects who abstained longer may have developed better strategies to control craving during acute abstinence, while still experiencing other withdrawal symptoms.

**Table 1:** Socio-demographic and clinical variables of the three groups at baseline. Data are reported as means (standard deviations) if not otherwise specified. *BDI* Beck depression inventory, *BAI* Beck anxiety inventory, *SHAPS* Snaith-Hamilton pleasure scale, *TAS-20* Toronto Alexithymia Scale, *ASRS* ADHD symptoms rating scale, *WAIS* Wechsler Adult Intelligence Scale, *FTND* Fagerstrom Test for Nicotine Dependence, *DBW* decision balance worksheet.

	Completers *N* = 19	Dropouts *N* = 34	Controls *N* = 31	*p*-value
Demographic data				
Age	37.7 (10.3)	41.4 (12.0)	37.6 (9.16)	0.300
Gender (n, %):				0.642
Female	7 (36.8%)	13 (38.3%)	12 (38.7%)	
Male	12 (63.2%)	21 (61.7%)	19 (61.3%)	
Race (n, %):				0.311
AfricanAmerican	7 (36.8%)	20 (58.8%)	19 (61.3%)	
Other	3 (15.8%)	2 (5.8%)	1 (3.22%)	
Caucasian	9 (47.4%)	12 (35.4%)	11 (35.5%)	
Psychometric measures				
BAI	3.37 (4.95)	1.76 (2.78)	1.30 (2.60)	0.100
BDI	3.63 (6.05)	4.11 (6.16)	2.23 (4.97)	0.406
BDI anhedonia	0.84 (1.30)	0.73 (1.22)	0.30 (0.79)	0.171
SHAPS	0.47 (1.12)	1.03 (1.95)	0.40 (0.72)	0.171
History of depression (n, %)	2 (10.5%)	2 (5.88%)	1 (3.33%)	0.715
TAS-20-Total Score	37.2 (8.85)	38.2 (11.1)	35.6 (8.22)	0.553
ASRS-Scale A	9.42 (4.93)	8.70 (5.10)	6.90 (5.38)	0.197
ASRS-Scale B	8.79 (4.70)	7.57 (5.45)	5.50 (4.71)	0.071
ASRS-Total Score	18.2 (8.71)	16.3 (9.73)	12.4 (9.64)	0.089
WAIS-Full IQ Score	102 (15.9)	99.5 (11.1)	100 (9.97)	0.753
Nicotine addiction				
FTND	4.32 (1.73)	4.63 (2.00)	-	0.805
DBW balance	-6.68 (7.41)	-5.75 (10.3)	-	0.725
Age start smoking	19.6 (5.85)	19.0 (3.94)	-	0.619
N. cigarettes per day	14.3 (5.56)	13.8 (5.24)	-	0.736

### Model-free variables did not change across sessions

**Fig. 3:**
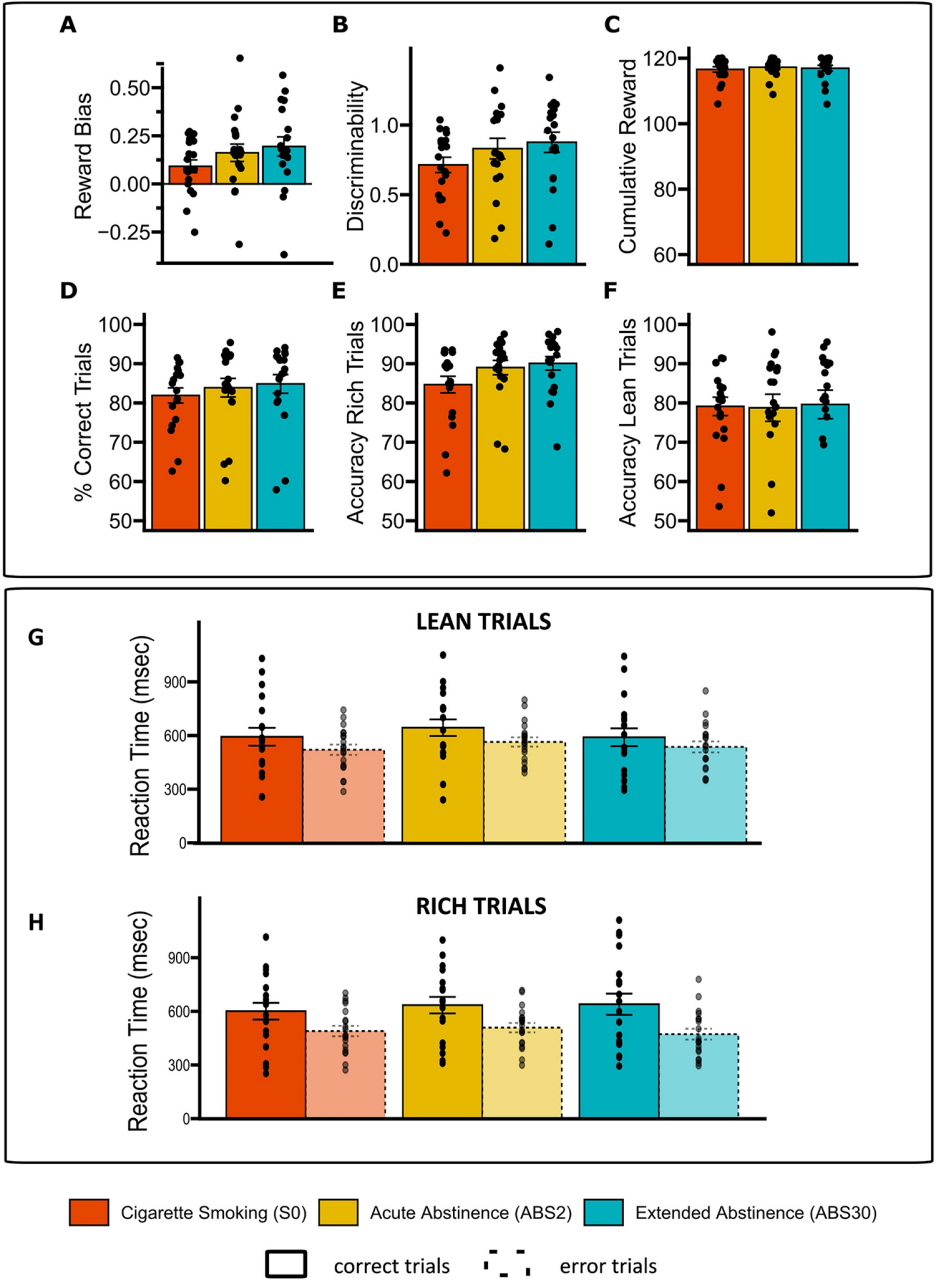
Signal Detection Analyses. Top panel: Mean reward bias (**A**), discriminability (**B**), cumulative reward (**C**), fraction of correct trials (**D**), accuracy for rich trials (**E**) and accuracy for lean trials (**F**) in completers by smoking status. Bottom panel: Mean reaction time for lean and rich trials by accuracy and smoking status. Error bars represent standard errors.

 No changes in reward bias (RB), discriminability (DR), cumulative reward (CR), and task accuracy (FR, AR and AL) were observed in completers (RB: F = 2.515; *p* = 0.118; DR: F = 2.527; CR: F = 0.52, *p* = 0.82; *p* = 0.118; AR: F = 3.338, *p* = 0.071; AL: F = 0.019, *p* = 0.89; FR: F = 0.79, *p* = 0.378), suggesting that a signal-detection approach was not sensitive enough to detect time-dependent changes in abstinent smokers without mood-related comorbidities (Fig. 3, Table S2). Also, there were no effects of sessions (F = 0.003, *p* = 0.9) or condition (F = 0.5, *p* = 0.5) on RT, although we observed a significant effect of accuracy on RT (F = 18.9, *p* < 0.01) with completers answering faster to correct vs. error trials (Fig. 3G).

### Comparing models: smokers shape decision-making on ‘Punishment’ and ‘Action-only’ learning strategies

Two of the five models—‘*Punishment*’ and ‘*Action*’—performed better than the others, although the difference between them was small (difference in the integrated Bayesian information criterion (ΔiBIC) = 2.1, Fig. S5) and insufficient to discriminate between the two^27^ (see Supplementary Results).

Briefly, the *Action* model assumes individuals learn from the value of each action, without pairing stimuli and actions effectively. This means participants simply learn and form expectations based on immediate rewards and disregard factors beyond the value of their own actions (see Prediction Error (PE) equation, Eq. 1). This model includes four parameters (α, β_reward_, Q_0_, γ).

1 $$\:PE=\left({\beta\:}_{reward}*\:{R}_{t}\right)\:{-\:Q}_{t}\left({a}_{t}\right)$$

The *Punishment* model, on the other hand, assumes subjects learn from the paired stimulus-action and weigh the trials on which no reward is given as punishing, treating the absence of rewards as aversive. This model includes five parameters (α, β_reward_, β_punishment_, Q_0_, γ) (see Eq. 2).

2 $$\:PE=\left({\beta\:}_{reward}*\:{R}_{t}\right)+\left({\beta\:}_{punishment}*\:{(1-R}_{t})\:\right)\:{-\:Q}_{t}\left({a}_{t},{s}_{t}\right)$$

### Punishment sensitivity is the only parameter that increased over time in successfully abstinent smokers

**Fig. 4:**
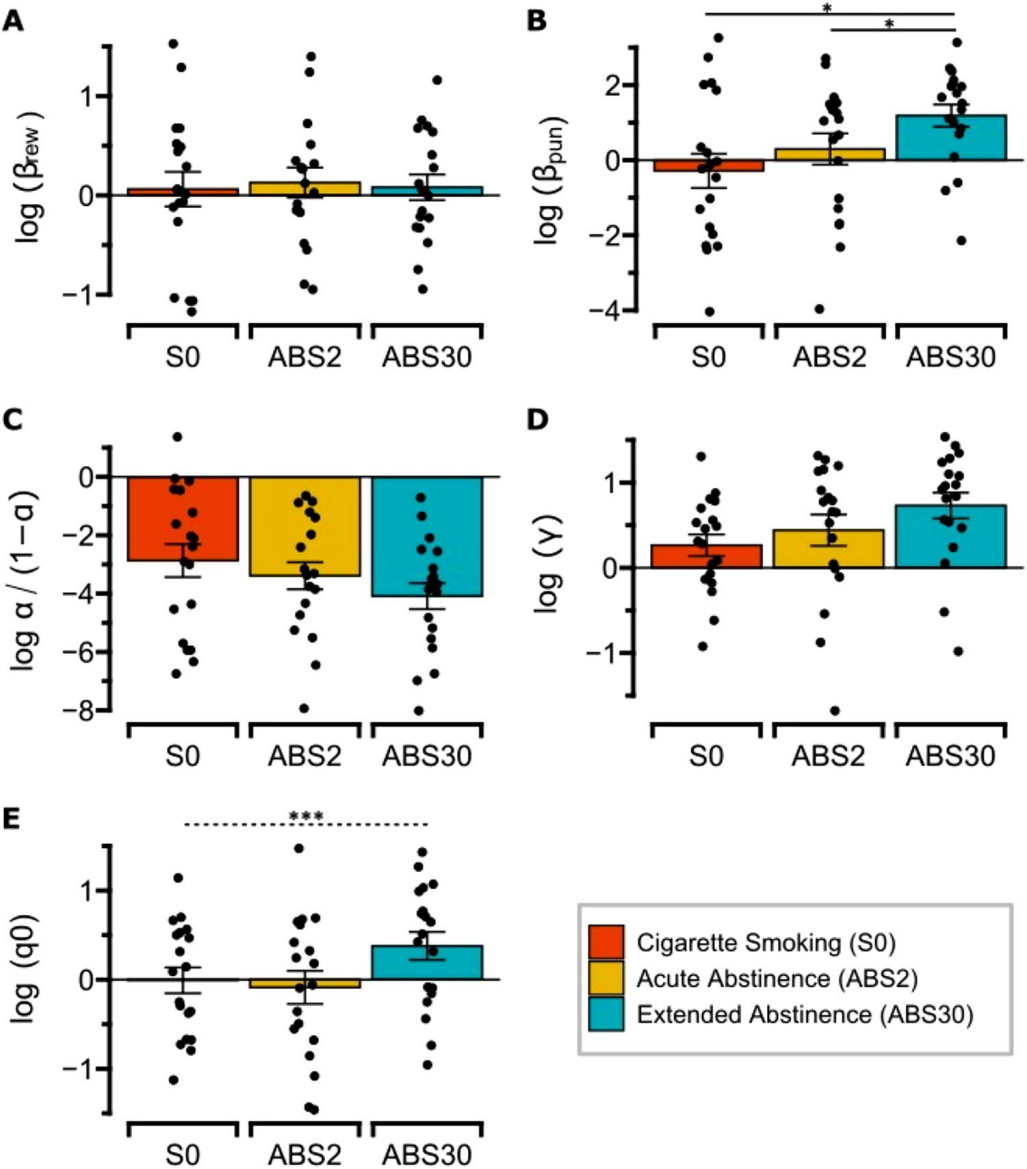
‘Punishment’ model output. Mean punishment sensitivity (log(β_punishment_), (**A**), reward sensitivity (log(β_reward_), (**B**), learning rate (log(α/(1-α)), (**C**), instruction sensitivity (log(γ), (**D**) and initial bias (log(q0), (**E**) in completers by smoking status. Error bars represent standard errors. Black bars identify the significant differences (*p < 0.05, ** *p* < 0.01, *** *p* < 0.001) at pairwise comparison when the ANOVA is significant. A dashed bar suggests that the difference does not survive when controlling for the others model’s parameters in the ANOVA.

Repeated measures ANOVAs were performed to compare model parameters across sessions (S0, ABS2, ABS30). A significant effect of sessions on punishment sensitivity was observed in completers (F = 7.142, *p* = 0.01), an effect that remained after controlling for the other parameters (F = 3.73, *p* = 0.041). Post-hoc analyses revealed that smokers were more sensitive to losses at the last session, after 30-day extended abstinence, compared to both baseline and acute nicotine withdrawal (S0 vs. ABS30, *p* = 0.032; ABS2 vs. ABS30, *p* = 0.042) (Fig. 4A, Table S3). In contrast, reward sensitivity, learning rate and initial bias did not change across sessions (log(β_reward_): F = 0.0009, *p* = 0.975; log(α/(1-α)): F = 3.064, *p* = 0.086; log(Q_0_): F = 3.6, *p* = 0.063) (Fig. 4B–D). While an effect was observed on instruction sensitivity (F = 4.64, *p* = 0.036), with log(γ) being increased at ABS30 vs. S0 (*p* < 0.0001) (Fig. 4E), this effect did not survive after controlling for the other parameters and may thus be interpreted as a potential learning effect due to task repetition (i.e., participants become more efficient in PRT performance over time because they are more familiar with the instructions and actions). Together, these results suggest that prediction errors differentially varied over time as a function of their valence (positive vs. negative). And, considering the effects on both reward and punishment sensitivity, smokers’ ability to learn from negative consequences seemed to be more affected than learning from rewarding experiences. Notably, completers and dropouts did not show difference in any of these parameters across abstinence, with no Session X Group interaction (log(β_punishment_): F = 0.945, *p* = 0.0.333; log(β_reward_): F = 0.029, *p* = 0.865; log(α/(1-α)): F = 0.421, *p* = 0.518; log(γ): F = 1.088, *p* = 0.299; log(Q_0_): F = 0.007, *p* = 0.933). Finally, completers did not differ from either dropouts or controls at baseline (log(β_punishment_): F = 1.272, *p* = 0.286; log(β_reward_): F = 0.495, *p* = 0.612; log(α/(1-α)): F = 1.298, *p* = 0.279; log(γ): F = 0.622, *p* = 0.539; log(Q_0_): F = 0.114, *p* = 0.539), suggesting the absence of a predetermining differences in parameters that may have biased results see Fig. S6.No effect of sessions on the Action model’s parameters. In contrast, for the Action model, reward sensitivity, learning rate, instruction sensitivity and initial bias did not change over time among completers (log(β_reward_): F = 0.211, *p* = 0.648; log(α/(1-α)): F = 0.276, *p* = 0.601; log(γ): F = 1.254, *p* = 0.268; log(Q_0_): F = 0.015, *p* = 0.902) (see Table S4). However, post-hoc analyses showed an increase in instruction sensitivity (log(γ)) at ABS30 vs. S0 (*p* = 0.008), suggesting an effect of task repetition, similar to the one observed with the ‘Punishment’ model. Details on model-based parameters for both the punishment and the Action model in dropouts and controls are detailed in Supplementary Results.

### The severity of nicotine addiction and craving play a role in punishment models parameter changes

 After observing that only the punishment sensitivity parameter from the “Punishment model” varied across sessions among completers, we then explored potential association with the subjective measures collected at baseline (FTND and DBW) and at each time-point (WSWS and TCQ). Also, given extant literature suggesting a key role for anhedonia and mood disorders in abstinence-induced reward processing alterations, we explored the association between RL parameters and the WSWS subscale for depressive symptoms. Lastly, given the potential role of attentional deficits in PRT performance, we explored the association of instruction sensitivity, reflecting subjects’ ability to follow instructions and recognize the presented stimuli, and other RL parameters with the WSWS subscale for concentration difficulty. Further details about the association between subjective measures of craving and withdrawal, model-based parameters and model-free variables are available in Supplementary Results.

**Fig. 5:**
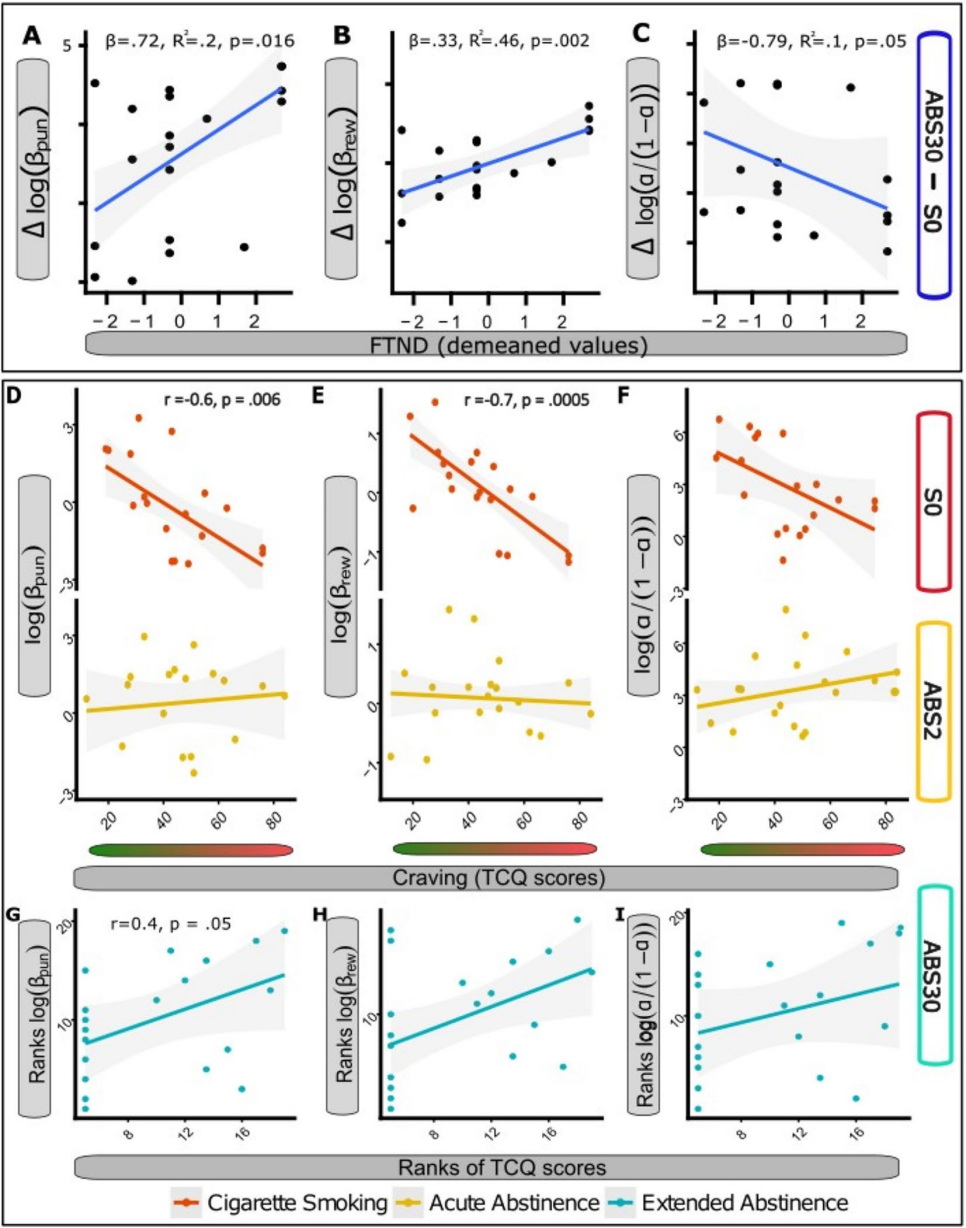
Relationship between smoking behavior and ‘Punishment’-based decision making. Top panel: change in Punishment Sensitivity (Δlog(β_punishment_), (**A**), Reward Sensitivity (Δlog(β_reward_), (**B**), and Learning Rate (Δlog(α/(1−α)), (**C**) in completers during extended abstinence (ABS30-S1). Smokers more severely addicted to nicotine (higher FTND) showed a greater increase in the parameters of interest. Bottom Panel: Correlation plots displaying relationships among Punishment Sensitivity (log(βpunishment)), Reward Sensitivity (log(βreward)), and Learning Rate (log(α/(1-α))) with Craving (TCQ scores) in completers, categorized by smoking status. Correlations at S0 and ABS2 were assessed using Pearson’s correlation (**D**–**F**), while correlations at ABS30 were evaluated using Spearman’s rank correlation (**G**–**I**).

The baseline severity of nicotine addiction (FTND) was inversely related to changes in punishment sensitivity (β = 0.72, R2 = 0.2, *p* = 0.016; Fig. 5A), reward sensitivity (β = 0.33, R2 = 0.46, *p* = 0.002; Fig. 5B) and learning rates (β = − 0.79, R2 = 0.11, *p* = 0.053; Fig. 5C) between S0 and ABS30 (ABS30-S0). No associations were found between the change in punishment sensitivity and the motivation to quit (DBW) (see Supplementary Results). Repeated-measures correlations were conducted to examine the relationships between PRT performance and subjective measures of withdrawal throughout abstinence. Interestingly, within-subject repeated-measure correlations were observed between Punishment model’s parameters and craving in completers (log(β_punishment_): *r*= − 0.43, *p* = 0.006; log(β_reward_): *r*= − 0.32, *p* = 0.045; log(α/(1-α)): *r*= − 0.3, *p* = 0.06). Notably, when separately evaluated at each timepoint, a negative correlation was observed between model-based parameters and craving in completers at S0 (log(β_punishment_): *r*= − 0.59, *p* = 0.006; log(β_reward_): *r*= − 0.71, *p* = 0.045; log(α/(1-α)): *r*= − 0.51, *p* = 0.06) (Fig. 5D–F). This correlation was not observed in completers at ABS2. These findings suggest that sated smokers perceiving stronger craving tend to be less sensitive to both rewards and losses, although this relationship seems to vary depending on the smoking status and individual characteristics.

Finally, the relationship between craving and model’s parameters tended to be reversed at ABS30 when TCQ measures showed a trend towards a positive association with log(β_reward_) and log(β_punishment_) (Spearman’s *r* = 0.40, *p* = 0.09; Spearman’s *r* = 0.45, *p* = 0.053, respectively). This suggests a switch in the behavioral response to craving during extended abstinence and may reflect a change in the way smokers perceive and respond to craving cues after prolonged abstinence.

Notably, no relationship between abstinence-induced depressive symptoms and punishment or reward sensitivity was observed. Considering that these subjects did not report symptoms of depression (see Table 1) and only 2 out of 19 reported previous depressive episodes, these results seem to align with previous evidence that reward processing alterations in abstinent smokers are more evident in vulnerable subjects with pre-existing baseline symptoms of depression^28^. However, larger samples are needed to better understand the impact of mood disturbances on decision-making process during nicotine abstinence.

Furthermore, no associations between abstinence-induced concentration difficulties and model parameters were observed. While the PRT is a probe of reward learning and does not directly evaluate attentional processing, these results are in line with the observation that neither instruction sensitivity nor discriminability rate differed across timepoints or between groups, and suggest that the observed heightened sensitivity to punishment, and task performance in general, does not depend on abstinence-induced concentration difficulties or attentional deficits.

## Discussion

Expanding upon previously reported reinforcement learning alterations following acute abstinence, we present new findings that demonstrate changes in reward-based decision-making during a biochemically-confirmed, 30-day extended nicotine abstinence (ABS30) in a cohort of nineteen treatment seeking smokers. Notably, this is also the first study to apply RL computational models to the PRT in smokers. While the findings may be limited by the relatively small sample size, they offer novel insights into the underlying mechanisms influencing decision-making during both acute and extended abstinence.

Contrary to our preregistered hypothesis that reward sensitivity and learning rate would be impaired as a function of duration of abstinence, punishment sensitivity emerged as the only parameter, among the five compared models, that changed during the smoking cessation protocol. Unfortunately, the absence of equally distributed, longitudinal measures in the control groups precludes definitive conclusions regarding the exclusive role of nicotine abstinence in driving these changes. However, while it is plausible that some changes in task performance could be attributed to task repetition effects, the specificity of the observed alterations, particularly in the context of the association between craving and decision-making parameters, suggests a more nuanced interpretation. In fact, we observed a clear impact of NWS on RL, and a dynamic relationship between craving and reward and punishment sensitivity over time, suggesting a significant recalibration of cognitive processes during abstinence. Specifically, while higher craving levels were associated with lower punishment and reward sensitivity and learning rates during ad libitum smoking at baseline, participants experienced a notable reduction in craving levels alongside an increase in punishment sensitivity following successful extended abstinence.

Although larger studies are needed to confirm the observed changes in punishment sensitivity following extended abstinence, the heightened sensitivity to negative outcomes observed during the last session (ABS30) compared to the previous ones (S1 and ABS2), may be interpreted as a cognitive adaptation aimed at fostering long-term abstinence. People with SUDs face challenges in appropriately assigning values to actions and learning the relationship between actions and outcomes^7,29^. They tend to prioritize positive rewarding experiences over negative consequences, and recent actions over past experiences^7,29^. Additionally, during acute abstinence, NWS and craving can skew perception, making negative outcomes less surprising and hindering learning from negative information^30^. The enhanced sensitivity to losses may help smokers correctly weigh the negative consequences of cigarettes (e.g., cancer, cardiovascular diseases, etc.) and restore a correct representation of reality, increasing their ability to make adequate predictions and guide actions toward rewards and away from harm. Possibly, this cognitive adaptation would likely result in behavioral adjustments aimed at minimizing losses and maximizing overall outcomes, including changes in risk-avoidance, vigilance and attentional focus, and adaptation to feedback, enabling more cautious but adaptive decision-making strategies in uncertain environments.

However, it is essential to acknowledge that the PRT does not adequately manipulate punishment, as missed rewards are treated as a form of punishment in our model, and the observed heightened punishment sensitivity does not necessarily equate to avoidance behavior, as emphasized by Chakroun et al.^31^. Moreover, as noted in the original work by Pessiglione et al.^32^, the effects of dopamine in reward and punishment learning can differ based on the context of losses versus the absence of rewards. This nuance is further illustrated by Chakroun et al.^31^, which indicates that interpreting the absence of reward as punishment might be less straightforward, particularly in humans. Thus, the generative model that participants infer while performing a task may significantly impact their learning process.

Contrasting task performance between subjects with high and low punishment sensitivity who were otherwise similar in terms of model’s parameters, we did not observe any difference in terms of cumulative rewards. However, subjects with higher punishment sensitivity demonstrated greater trial-by-trial accuracy for both lean and rich trials that was maintained over trials (see Fig. S8). This supports our hypothesis that an increased punishment sensitivity may lead subjects to avoid missed rewards, resulting in a more precise behavior in response to negative feedback. At the same time, this enhanced accuracy will not alter task performance in terms of reward and can be interpreted as a greater responsiveness to suboptimal choices.

These dynamics highlight the need for further investigation to understand how punishment sensitivity and behavioral outcomes may vary across different contexts and frameworks. Exploring various tasks with alternative definitions of punishment could help to better delineate punishment sensitivity, particularly in terms of sensitivity to losses. Finally, larger longitudinal studies are essential to clarify the role of prolonged nicotine absence in the observed effects, particularly given the limitations associated with a lack of repeated assessments in dropouts and healthy controls within our protocol.Notably, the PRT we employed does not include explicit smoking cues, and as such, any bias observed in this task is more likely to reflect the participants’ intrinsic cognitive strategies, rather than external motivational influences. While our approach aimed to understand how these cognitive strategies vary during nicotine abstinence, it is important to note that neither the task nor the study design were specifically intended to invoke NWS symptoms, contrary to that observed with tasks in which specific cues are intended to induce craving and related behavioral responses (e.g., cue-reactivity tasks^33^). In this context, our findings suggest that the decision-making process may change across abstinence, potentially pushing individuals away from certain strategies (e.g., reward-focused strategies) and towards others (e.g., heightened punishment sensitivity). This shift may represent an adaptive response to the multiple challenges of abstinence, even if it does not directly involve motivational biases related to smoking cues. Although we cannot rule out possible RL adaptations to external motivation, the observed differences in punishment sensitivity during abstinence and the observed correlations with FTND seem to suggest that RL in abstinent smokers varies with the severity of nicotine addiction and the presence (or absence) of nicotine. Future research could benefit from integrating both motivational and cognitive perspectives by using tasks that capture both intrinsic problem-solving strategies and cue-driven biases, in order to better define the interaction between these two key components of nicotine addiction.

In addition to motivational aspects, concentration difficulties and attentional deficits are key component of NWS that may exert an impact on PRT performance. Although the PRT is a probe of reward-based learning and does not directly evaluate attentional functions^34^, it is important to note that both the signal detection analysis and the RL models consider the ability to discriminate between two stimuli and follow instructions. The signal detection analysis revealed no differences in discriminability rates between smokers and controls, suggesting that basic attentional processing remained intact. Similarly, when using RL models, we found no differences in instruction sensitivity between smokers and controls at baseline. This parameter is crucial as it reflects participants’ adherence to task rules, further implying that smokers did not exhibit attentional deficits in this context. Additionally, a detailed analysis of reaction times—often used as an indirect measure of cognitive function in tasks requiring discrimination between similar stimuli^35^—did not show any group differences. Although a specific test targeting executive and cognitive functions was not conducted, these findings collectively suggest that the heightened sensitivity to punishment observed herein is unlikely to be driven by concentration difficulties or attentional impairments.

While changes in decision-making strategies were captured by the RL model, no changes in task performance or model-free variables were observed. These results did not replicate at least one previous study that showed nicotine withdrawal-induced blunting of reward learning at the PRT^12^. Although this may reflect interindividual differences in smokers that may require larger samples to be investigated, the difference in these results may be interpreted in light of previous evidence showing nicotine-withdrawal effects on RL to be more pronounced in individuals with Major Depressive Disorder (MDD)^15,36,37^ or vulnerable to mood disorders^38^. In fact, while the current study investigated the effects of 48-h and 30-day abstinence in non-depressed smokers, the diminishing effect on PRT reward bias was previously observed in smokers, 57% of whom reported symptoms of a Major Depressive Disorder, after 24-h withdrawal. This interpretation also aligns with recent research involving smokers with current or past MDD undergoing a 12-week smoking cessation protocol^28^. In this study, regardless of the treatment received (standard behavioral cessation treatment vs. behavioral activation integrated with standard treatment, combined with varenicline vs. placebo), a reduction in reward bias during treatment was noted in smokers with high symptoms of depression, while no changes in reward bias were observed in smokers with medium or low symptoms of depression^28^. Although the timing of the assessments does not allow a direct comparison of our findings with those of Gollan et al.^28^, it is noteworthy that repeated assessments of the PRT during a smoking cessation protocol did not reveal any specific effect linked to task repetition and learning. Also, the absence of any specific effect associated with treatment groups^28^ suggests the PRT may be more sensitive to abstinence than specific treatments or treatment load. Larger studies in subjects undergoing smoking cessation, with repeated measures in both completers and dropouts, may help disentangle the observed effects and allow causal interpretations.

In addition to the ‘Punishment’ model, a second model, named ‘Action only’, also fit the data. This model assumes subjects do not learn from the stimuli but *only* from the value of each action, so that they are unable to pair stimuli and actions effectively. Although we did not observe any change of its parameters, this result is noteworthy. First, this model suggests subjects were disregarding factors beyond the immediate actions and rewards. Although this decision-making strategy may simplify the learning process in a static context, where stimuli do not change and reward probabilities associated with each choice remain stable over time, individuals who do not learn from stimuli might face challenges in adapting their behavior to new circumstances in dynamic environments where the value of both stimuli and actions change over time. This is particularly relevant in the context of nicotine addiction and the dynamic challenges that smokers face during abstinence. Although the exploratory nature of these findings limits our ability to draw any clinical implication, it is also important to notice that interventions or training programs might be developed to improve smokers’ ability to integrate information from both stimuli and actions, thus increasing adaptability.

The study has several important strengths: the well-structured, within-subject, longitudinal design and the incorporation of medium-term (30 days) biochemically verified abstinence effects on decision-making and reinforcement learning, which provide, for the first time, a more nuanced and complete picture beyond acute abstinence, when most individuals return to smoking. Moreover, the analytical strategies (i.e., pre-registration) were implemented to increase reliability and meaningfulness of the observed effects^39^.

However, other design and analytical limitations should also be mentioned, including that participant served as their own control in longitudinal observations and the absence of a longer, more clinically relevant time point following smoking cessation. In a large, longer smoking-cessation protocol probing reward learning with PRT at week 1, 7 and 14 after quitting, Gollan et al.^28^ showed a decreased response bias at week 14 compared to previous timepoint. Although this effect was only present among smokers with high baseline depression symptoms, these findings underscore the importance of extending follow-up periods beyond ABS30. The heightened punishment sensitivity observed at ABS30 might represent a distinct phenomenon that could evolve differently at longer follow-ups.

Additionally, the main analyses were conducted on those subjects who underwent evaluation at ABS30 (*n* = 19). This relatively small number of successful quitters may have impacted the power and sensitivity to detect certin effects, particularly those related to RL parameters and their relationship with withdrawal symptoms. Moreover, the small sample size limits our ability to perform further analyses that could disentangle the effects of sex and age on the observed findings. Consequently, our findings should be interpreted with caution, and their generalizability await future studies with larger samples to validate these findings and explore their broader applicability. Finally, the absence of longitudinal measures in the control groups do not allow us to exclude that the observed changes in RL model’s parameters are due to factors other than nicotine abstinence, such as task practice effects and the effects of CBT and motivational interviewing incorporated into the treatment regimen, which itself may have had an effect in increasing punishment sensitivity, raising awareness for negative consequences and changing the relative values of cigarettes. While previous findings from randomized clinical trials about CBT effects on decision-making process are non-homogeneous^40^ and the current design does not allow to better define the specific mechanism(s) underlying the observed changes in RL during smoking cessation, it is noteworthy that completers in the current study underwent only a few sessions of motivational training (an average of 3^2–7^ at the time of the assessment). Larger studies that incorporate repeated measures of RL regardless of smoking status may help determine whether the observed RL effects are the results of the pharmacological manipulation, and the allostatic changes induced by the absence of nicotine.

While there is an extensive literature supporting the use of PRT in clinical population, only a few studies evaluated its psychometric properties in a within-subjects design, i.e. most studies using repeated PRT assessments, evaluated task performance over a brief period of time^16,25,41^ and/or used different analysis approaches^16,41^. Finally, although recent evidence from a smoking cessation protocol^28^ suggest no specific effect linked to task repetition and learning, they only applied a model-free approach and we thus cannot exclude that a more sensitive computational approach is capturing test-retest effects.

In conclusion, we present unique evidence of changes in decision-making strategies in smokers who successfully abstained from cigarettes for 30 days compared to pre-quitting satiety. We observed heightened punishment sensitivity following successful extended abstinence compared to both ad libitum smoking and acute abstinence, in the presence of preserved reward sensitivity. Moreover, we observed pronounced changes in the associations between both punishment and reward sensitivity and craving, suggesting a nuanced interplay between these variables throughout the smoking cessation protocol. Our interpretation is that the heightened punishment sensitivity may indicate a greater awareness of negative consequences, enabling individuals to recognize and confront factors that act as punishers or predictors of punishment (such as lung cancer, social isolation, etc.) to which they were desensitized by smoking. Further studies may help clarify the mechanisms underlying the increased punishment sensitivity and its role in nicotine abstinence in the context of a unified model of decision-making.

## Electronic supplementary material

Below is the link to the electronic supplementary material.

Supplementary Material 1

## Data Availability

The data that support the findings of this study are not openly available due to reasons of sensitivity and are available upon reasonable request. Requests should be addressed to tross@nih.gov.
