# Supplementary material for: Longitudinal changes in reinforcement learning during smoking cessation: a computational analysis using a probabilistic reward task
Source: Sci Rep. 2024 Dec 31;14:32171. doi: 10.1038/s41598-024-84091-y (PMC11688494; doi:10.1038/s41598-024-84091-y)
Supplement: Supplementary file 1 — Supplementary Material 1 [file 41598_2024_84091_MOESM1_ESM.docx]

**Supplementary Information**

[1. Supplementary Methods 1](#_Toc164624985)

[1.1. Supplementary methods for data collection 1](#_Toc164624986)

[1.2. Supplementary methods for model-free analyses 2](#_Toc164624987)

[1.3. Supplementary methods for model-based analyses 4](#_Toc164624988)

[1.3.1. Models’ description 4](#_Toc164624989)

[1.3.2. Model fitting 8](#_Toc164624990)

[1.3.3. Model comparison 8](#_Toc164624991)

[1.3.4. Model validation 9](#_Toc164624992)

[1.4. Statistical methods for analysis 9](#_Toc164624993)

[2. Supplementary Results 10](#_Toc164624994)

[2.1. Socio-demographic and clinical variables at baseline 10](#_Toc164624995)

[2.2. Nicotine Withdrawal Syndrome 10](#_Toc164624996)

[2.2. Model-free results 11](#_Toc164624997)

[2.3 Model-based results. 15](#_Toc164624998)

[2.3.1 Model fitting and model selection 15](#_Toc164624999)

[2.3.2 Model-based parameters 17](#_Toc164625000)

[2.4 Association between subjective measures of craving and withdrawal, ‘Punishment’ parameters and model-free variables. 20](#_Toc164625001)

[2.5 Association between subjective measures of craving and withdrawal, ‘Action-only’ parameters and model-free variables. 23](#_Toc164625002)

[3. References 24](#_Toc164625003)

# Supplementary Methods

## Supplementary methods for data collection

To be eligible for the study, participants were required to be right-handed, aged 18–55 years, free of active drug or alcohol abuse/dependence (other than nicotine dependence in smokers), report no current psychiatric or neurological diagnosis, and present no contraindications for magnetic resonance (MR) imaging. Upon arrival for their first MR scanning session, all participants underwent a medical assessment, including screening for symptoms of depression (Beck Depression Inventory (BDI) (1)) and anxiety (Beck Anxiety Inventory (BAI) (2)). Also, questionnaires to evaluate the presence of alexithymia (Toronto Alexithymia Scale (TAS-20) (3)) and ADHD (ADHD Symptoms Rating Scale (ASRS) (4)) were administered.

Urine test (ASC-11, American Screening Corp., Shreveport, LA) for recent usage of illicit drugs (opiates, oxycodone, benzodiazepines, buprenorphine, cocaine, amphetamines/methamphetamines, tetrahydrocannabinol (THC), methadone, phencyclidine, and methylenedioxymethamphetamine) and an alcohol assessment (Alcohol sensory IV, Intoximeters Inc., St. Louis, MO) were evaluated at each session. Intoxication was exclusionary, requiring scan rescheduling.

## Supplementary methods for model-free analyses

In order to compare our sample to previous studies, we applied signal-detection analysis methods (1, 2). Based on the randomization in the pairing between the stimuli (short vs. long) and the probability of getting a reward, the stimuli were classified as *rich* and *lean*, and six behavioral variables of interest were calculated:

- *Reward Bias* (RB), defined as the individual’s responsiveness to rewards, detecting the tendency of a subject to report the rich compared to the lean stimuli.
- *Discriminability Rate* (DR): defined as the individual’s ability to discriminate between the two stimuli.
- *Fraction Correct Responses* (FR): overall participants’ accuracy expressed as the percentage of correct responses.
- *Cumulative reward (CR):* defined as the sum of all rewards received by the subject, reflecting individual’s task performance.
- *Accuracy for rich stimuli* (AR) and *lean stimuli* (AL), reflecting the ability to identify each of the two stimuli correctly.

For each subject we also computed the mean *Reaction Time (RT)* for rich and lean stimuli, based on the accuracy of the response (correct responses for rich stimuli, correct responses for lean stimuli, errors for rich stimuli and errors for lean stimuli).

Reward Bias (RB) and discriminability rate (DR) were computed as following:

$$RB= \frac{1}{2} log\left( \frac{\sum_{1}^{t} {Rich}_{correct}\sum_{1}^{t} {Lean}_{incorrect}}{\sum_{1}^{t} {Rich}_{incorrect}\sum_{1}^{t} {Lean}_{correct}} \right)$$

Equation (1)

$$DR= \frac{1}{2} log\left( \frac{\sum_{1}^{t} {Rich}_{correct}\sum_{1}^{t} {Lean}_{correct}}{\sum_{1}^{t} {Rich}_{incorrect}\sum_{1}^{t} {Lean}_{incorrect}} \right)$$

Equation (2)

where Σ Rich is the number of times, out of all the trials (t), in which the participants faced the more rewarded stimulus. Similarly, Σ Lean define the sum of trials in which the participants faced the less rewarded stimulus. The subscript refers to the correctness of participants’ response so that, for example, Σ Rich _correct_ is the number of trials in which the participant faced the more rewarded stimulus and identified it correctly.

Accuracies for rich (AR) and lean (AL) stimuli refers to the correctness of a participants’ response.

AR, for example, was computed as:

$${Accuracy}_{Rich}=\frac{\sum_{1}^{t} {Rich}_{correct}}{\sum_{1}^{t} Rich}$$

Equation (3)

Abnormally short (< 500 ms or -3 standard deviations) and long (> 1500 ms or +3 standard deviations) reaction time (RT) trials were excluded. After this cleaning procedure, 3.19% of the original 63,300 trials we excluded, leaving 61,279 trials for final analyses.

## Supplementary methods for model-based analyses

Current advances in computational modelling in psychiatry allow for modelling learning and decision-making processing in greater detail. Recently, Huys et al (3) developed a set of reinforcement learning models that are able to account for every choice that a subject makes at every trial. Moreover, they developed a method to compare models (3), allowing for multiple model comparisons without losing trial-by-trial specificity. More specifically, for each participant, different models are applied and computed, and are compared to each other by performing fixed-effects empirical Bayesian batch inference. Statistical inference using the Bayes' theorem allows one to compute the probability for a specific event and update it every time new information is available, with the inference computed at a subject level. The method is performed by first fitting priors to a set of models, and then performing model comparison. Synthetic data are generated and compared to actual data with visual comparisons available.

Matlab scripts were made available from prof. Huys upon request and used to compute and then compare all available models (as described below). Scripts are available at: <https://github.com/mpc-ucl/emfit>. Model specifications for the probabilistic reward task are available at <https://quentinhuys.com/abstr_HuysEa13-MDDMetaAnalysis.html>.

### Models’ description

A standard reinforcement learning (RL) model was modified to capture the behavioral choices in the experiment. Choices were modelled with a softmax decision function that expresses selection probabilities as a sigmoidal function. This model assumes that the probability of choosing the action *a* (e.g., pressing the ‘Z’ key) given the stimulus *s* (e.g., long mouth), depends on the difference between its weight and the weight of the alternative choice ($\overline{a}$).

$$p(a_{t}|s_{t})=\frac{1}{1+e^{- (W_{t}\left( a_{t}, s_{t} \right)-W_{t}\left( \overline{a}_{t}, s_{t} \right))}}$$

Equation (4)

where *p* (a_t_|s_t_) denotes the probability of choosing the action *a* (e.g., pressing the key ‘Z’) when the stimulus s (e.g., long mouth) appears on each trial *t*.

*W* represents the choice weight assigned to each choice (actual *a* and alternative $\bar{a}$) when presented with stimulus *s* at trial *t*. The choice weights (W) changes over time as a function of (1) the ability to follow the instructions, defined by a first parameter namely instruction sensitivity (γ), and (2) the expected reward (Q).

$$W_{t}\left( a_{t},s_{t} \right)=\gamma*I\left( a_{t},s_{t} \right)+\zeta*Q_{t}(a_{t},s_{t})$$

Equation (5)

where the instructions *I* (a_t_, s_t_) defines the instructed choice *a* for the stimulus *s*. For instance, the instruction is coded as 1 when the participant has to press the key ‘Z’ when the long mouth appear, and 0 otherwise. The γ parameter thus determines the participants ability to follow the instructions. The greater the γ parameter, the more the instructed response contributes to the choice. The γ parameter can vary between 0 and 1.

*Q* represents the effect of the experienced rewards on previous trials. Of note, the experienced value is itself weighted on another parameter ζ, named beliefs, that represents participants’ average uncertainty about which stimulus was actually presented. The ζ can vary between 0 and 1 with greater values defining a greater certainty on the presented stimulus. The participants hence have a probability ζ that they had seen a stimulus *s* (e.g., long mouth) and a 1-ζ probability that they had seen the stimulus $\overline{s}$ (e.g., short mouth)

The expected reward (Q) is hence updated at each trial according to a prediction error, defined as the difference between the received (R) and the expected (Q) reward.

$$Q_{t+1}\left( a_{t},s_{t} \right)=Q_{t}\left( a_{t},s_{t} \right)+ \alpha*PE$$

Equation (6)

$$PE=\left( \beta* R_{t} \right){- Q}_{t}\left( a_{t},s_{t} \right)$$

Equation (7)

Where α represent a learning rate that can vary between 0 and 1. As the learning rate approaches 1, learning is so fast that the Q values are simply the last experienced outcome for each choice-action pair. A learning rate of 0, instead, means that the subjects are insensitive to each trial outcome and the only value Q that defines their choice weight (W) is their starting point Q_0_. This latter parameter, defined initial action bias, represent the expected reward at trial 0, that is at the beginning of the experiment. The Q_0_ parameter can vary between 0 and 1 with a value of 1 when the participant is expecting a reward from the very first trial. Values of 0.5 suggest that the participants are agnostic to the possible outcome (i.e., equal possibility of reward or not).

The prediction error (PE) here is defined as the difference between the reward at trial *t* (R_t_) coded as 0 for no reward and 1 for reward, and the expected value (Q) up to that given trial. The reward is weighted for a parameter β, named reward sensitivity, that is the value each participant assigns to the reward. The parameter β can vary between -1 and 1 and the larger the β, the more sensitive the participant is to a reward of a given magnitude, or the greater the internal worth of an external reward.

The model allows also for the possibility that subjects treat non-rewards as actual punishments. This assumes individuals correctly assign the rewards to particular stimulus-action combinations. The first term of the prediction error hence includes two sensitivities, one for the rewards and one for the punishment as follows:

$$PE=\left( \beta_{reward}* R_{t} \right)+\left( \beta_{punishment}* {(1-R}_{t}) \right) {- Q}_{t}\left( a_{t},s_{t} \right)$$

Equation (8)

Where R represent the reward, 1-R present the missed reward, or punishment, and each of them is weighted on a different sensitivity parameter β.

Specifically, based on previous literature (3), we adopted five different models that included the parameters described in the main text.

The five main models were:

- **Stimulus-Action model**. This basic stimulus-action Rescorla-Wagner model assumes individuals correctly assign the rewards to particular stimulus-action combinations. It has four parameters: a reward learning rate (α), a reward sensitivity (β), an instruction sensitivity (γ) and an initial action bias (Q_0_). In this model, choices are determined by the term in the softmax:

$$W_{t}\left( a_{t},s_{t} \right)=\gamma*I\left( a_{t},s_{t} \right)+Q_{t}(a_{t},s_{t})$$

Equation (9)

and the *Q* value of the chosen action *a* for the stimulus *s* is updated based on received rewards:

$$Q_{t+1}\left( a_{t},s_{t} \right)=Q_{t}\left( a_{t},s_{t} \right)+ \alpha*PE$$

$$PE=\left( \beta_{reward}* R_{t} \right) {- Q}_{t}\left( a_{t},s_{t} \right)$$

Equation (10)

- **Punishment model**. This model is similar to the previous one but also allows for the possibility that subjects treat non-rewards as actual punishments. To this aim it two separate sensitivities for reward and non-reward events. It hence has five parameters: a reward learning rate (α), two separate sensitivities, one for reward (β_reward_) and one for punishment -or lack of reward- (β_punishment_), an instruction sensitivity (γ) and an initial action bias (Q_0_). This means that, under this model, participants treat the outcome ‘no reward’ as a punishment, reducing the probability of the associated action, instead of treating it as a non-informative null outcome, as intended by the task design. In this model, choices are determined by the same term in the softmax:

$$W_{t}\left( a_{t},s_{t} \right)=\gamma*I\left( a_{t},s_{t} \right)+Q_{t}(a_{t},s_{t})$$

Equation (11)

but the *Q* value of the chosen action *a* for the stimulus *s* is updated based on both received and missed rewards:

$$Q_{t+1}\left( a_{t},s_{t} \right)=Q_{t}\left( a_{t},s_{t} \right)+ \alpha*PE$$

$$PE=\left( \beta_{reward}* R_{t} \right)+ \left( \beta_{punishment}* {(1-R}_{t}) \right) {- Q}_{t}\left( a_{t},s_{t} \right)$$

Equation (12)

- **Action model**. This model assumes individuals only learn about the value of each action, independent of the stimuli. It has four parameters: a reward learning rate (α), a reward sensitivity (β), an instruction sensitivity (γ) and an initial action bias (Q_0_). Note that although this model superficially has the same parameters of the stimulus-action model, the meaning of these parameters is different so that the stimulus (short vs long) is not taken into account and the participant do not discriminate between the two stimuli. In this model, choices are determined by:

$$W_{t}\left( a_{t} \right)=\gamma*I\left( a_{t} \right)+Q_{t}(a_{t})$$

Equation (13)

where the *Q* value of the chosen action *a*, regardless of the stimulus *s*, is updated based on rewards:

$$Q_{t+1}\left( a_{t} \right)=Q_{t}\left( a_{t} \right)+ \alpha*PE$$

$$PE=\left( \beta_{reward}* R_{t} \right)-\left( a_{t} \right)$$

Equation (14)

**Belief model**. Compared to the previous ones this model as a new parameter that capture the participants’ average uncertainty about which stimulus was actually presented (ζ). As subjects are unsure about the presented stimulus, they might assign rewards to both stimuli, with only a certain preference for the actually presented stimulus. The model has five parameters: a reward learning rate (α), a reward sensitivity (β), an instruction sensitivity (γ), an initial action bias (Q_0_), and a belief parameter (ζ). As ζ approaches 1, participants are certain about their choice and use the correct stimulus-action value to make their decision. In doing so, this model converges to the stimulus-action model, with choices determined by

$$W_{t}\left( a_{t},s_{t} \right)=\gamma*I\left( a_{t},s_{t} \right)+\zeta*Q_{t}(a_{t},s_{t})$$

Equation (15)

and the *Q* value of the chosen action *a* for the stimulus *s* updated based on received rewards (see Equation 10).

- **Counterfactual model**. As subjects are unsure about the presented stimulus, they might assign rewards to both stimuli, with only a certain preference for the actually presented stimulus. This model additionally performs counterfactual updates, i.e. learns about both stimuli on each trial, weighted by how likely they were to have been presented. The model has five parameters: a reward learning rate (α), a reward sensitivity (β), an instruction sensitivity (γ), an initial action bias (Q_0_), and a belief parameter (ζ).

Equation 5 that defines the choice weights (W), under this counterfactual model hence becomes

$$W_{t}\left( a_{t},s_{t} \right)=\gamma*I\left( a_{t},s_{t} \right)+\zeta*Q_{t}\left( a_{t},s_{t} \right)+(1-\zeta)*Q_{t}(a_{t},\overline{s}_{t})$$

Equation (16)

All the parameters entered in the analyses were represented as non-linearly transformed variables. Specifically all the parameters were log-transformed before any further analyses (e.g., reward and punishment sensitivities = log(β); instruction sensitivity =log(γ)), apart from the learning rate that is the logarithm of a further transformation (log(α/(1- α))).

### Model fitting

Each participant choice on each trial was fitted on the above-mentioned models. Here, we also considered the repeated measures within each subject as separate tasks, ending with n=211 tasks performed and n=63,300 choices made. Model fitting procedures are described in detail elsewhere (3). Briefly we adopted an Expectation-Maximization (EM) algorithm to estimate each participant’s parameter. The EM algorithm is an iterative approach that consist of two steps. In the first step the algorithm estimates the parameter (Expectation/estimation step, E-step). In a second step, the algorithm optimises the parameter so that it can explain the data more clearly (Maximization-step, M-step). These two-steps are repeated until the convergence of the parameter’s value occurs. Convergence is defined as the point at which the smallest difference in the probability of two variables is reached. That is when the parameters from the two steps are matched with each other. Specifically, here we used a Laplace’s approximation to find posterior probability distribution for each parameter for each participant.

### Model comparison

More complex models (i.e., with more parameters) might fit the data better but at the expense of overfitting. A way to assess model parsimony is the Bayesian model comparison. Each model hence has an assigned iBIC (for ‘integrated BIC’), that may be considered as the value by which that model is able to explain the data, considering the complexity. The iBIC approximates the model parsimony assessed by the integration of the posterior probability of each model on the entire dataset, the fitted priors for each individual, and the standard Bayesian information criterion (BIC) approximation at the group level. The difference between the iBIC values of two different models is an approximation of the models’ relative log Bayes factor, and it was used to select the best model describing the sample. Two key aspects of this quantity are that 1) it punishes overly complex models; 2) it assesses this at the group, rather than the individual level. The higher this number, the poorer the combination of model fit and model simplicity. In other words, the lower the iBIC, the better the model. Differences above 10 are typically considered to be strong evidence for one model over the other (4, 5).

### Model validation

After model comparison and selection, we first evaluated how good the model was in retrieving the behavioural data based on the stimuli presented, and then we explored the association between the winning model’s parameter and the task performance in the entire sample. Specifically, we tested the bivariate correlations between the model’s parameters and the five model-free variables of interest.

## Statistical methods for analysis

R (version: 3.6.3, R-project.org) was used for data mining, data visualization and statistical analysis. Except for the specific functions reported in the main manuscript, the following R-packages were used: *dplyr, tidyverse, compareGroups, rstatix, ggplot2, ggpubr, corr, rmcorr.*

Beyond the preregistered hypotheses, we also conducted the following analyses.

Moreover, given the structure of the task that consists in three separate blocks of 100 trials each, and given previous studies showing specific effects of time (6), two-way ANOVA with sessions (S0, ABS2, ABS30) and blocks (1, 2, 3) as within-subject factors was used to evaluate the changes in model-free variables during specific blocks.

Last, given the observed increased punishment sensitivity at extended abstinence vs. other timepoints, we investigated whether this effect was borne out in subjects’ behavior. To calculate the subjects’ sensitivity to losses, we therefore calculated the individual’s percentage of correct responses following a negative trial (a trial in which they did not receive any reward) for each block at each session. Two-way ANOVA with sessions (S0, ABS2, ABS30) and blocks (1, 2, 3) as within-subject factors were then applied to evaluate changes in this indirect measure of sensitivity to losses.

# Supplementary Results

## Nicotine Withdrawal Syndrome

Similarly to what observed in completers, subjective ratings of withdrawal were modulated by nicotine abstinence in dropouts (**Figure S1**). Notably when comparing between completers and dropouts, we observed Session X Group interaction on TCQ (F=5.051, p=0.027), such that dropouts, but not completers, experienced increased craving between S0 and ABS2 (p < 0.001). Conversely, WSWS showed a main effect of Session (F=12.78, p < 0.001) but no effects of Group nor Session X Group interaction (F=0.0004; p=0.982), such that withdrawal symptoms increased from baseline to acute abstinence in both dropout and completers. These results suggest that subjects who can abstain longer may have developed better strategies to control craving during acute abstinence, while still experiencing other withdrawal symptoms.


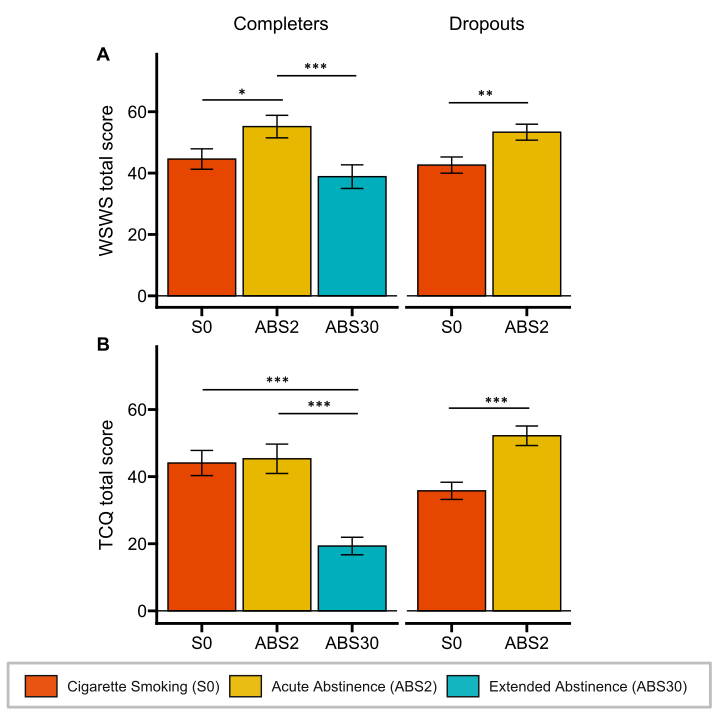


***Figure S1. Abstinence-induced changes in measures of craving and withdrawal.*** Mean self-report measures of nicotine craving (TCQ) (top panel) and withdrawal symptoms (WSWS) (bottom panel) by study group and session. Error bars represent standard errors. Black bars identify the significant differences (* = p<0.05, ** = p < 0.01, *** = P < 0.001) at pairwise comparison when the ANOVA was significant.

## Model-free results

Beyond the preregistered hypotheses, completers did not differ on response bias or other model-free variables across blocks (**Figure S2**). Also, no changes in reward bias (RB), discriminability (DR), task accuracy (AR and AL) and cumulative reward were observed in dropouts (RB: F=1.15; p=0.29; DR: F=0.26; p=0.61; AR: F=0.23, p=0.64; AL: F=0.94, p=0.34; CR = F=0.10, p=0.75) (**Figure S2**, **Table S2**). Completers and dropouts did not show any significant differences during abstinence, with no Session X Group interaction observed (RB: F=0.013; p=0.908; DR: F=1.613; p=0.207; AR: F=0.667, p=0.416; AL: F=0.295; p=0.558). Also, the model free variables did not differ between completers, dropouts, and controls at baseline (RB: F=0.912; p=0.406; DR: F=0.557; p=0.575; AR: F=0.26, p=0.772; AL: F=0.33; p=0.72) (see **Figure S3**).

Last, given the observed increased punishment sensitivity at extended abstinence vs. other timepoints in completers, we investigated whether this effect was borne out in subjects’ behavior. No significant effects of blocks or sessions were observed on this indirect measure of sensitivity to losses (see **Figure S4**).


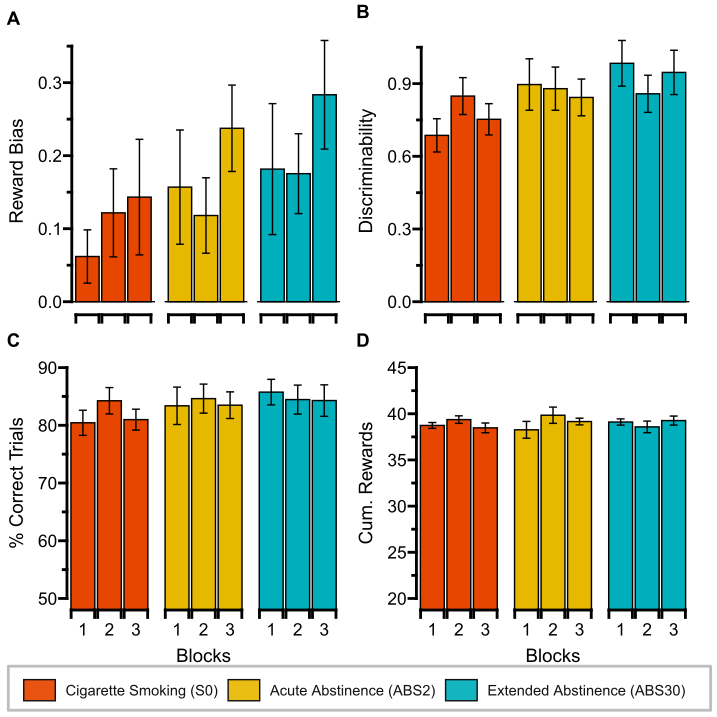


***Figure S2. Signal Detection Analyses.*** Mean reward bias (A), discriminability (B), correct responses (%, C) and cumulative rewards (D) by block and smoking status in completers. Error bars represent standard errors.

**Table S2. Model-free variables.** Mean values (standard deviations) by study group and smoking status.

|  | CONTROLS N: 31 | Completers N: 19 | | | DROPOUTS N: 34 | |
| --- | --- | --- | --- | --- | --- | --- |
|  | Baseline | Baseline | Acute Abstinence | Extended Abstinence | Baseline | Acute Abstinence |
| Reward bias | 0.17 (0.20) | 0.09 (0.15) | 0.17 (0.19) | 0.18 (0.22) | 0.10 (0.25) | 0.16 (0.20) |
| Discriminability | 0.78 (0.36) | 0.72 (0.24) | 0.85 (0.35) | 0.88 (0.32) | 0.81 (0.31) | 0.76 (0.32) |
| Accuracy Lean Trials | 0.77 (0.15) | 0.79 (0.10) | 0.79 (0.15) | 0.80 (0.16) | 0.80 (0.14) | 0.77 (0.16) |
| Accuracy Rich Trials | 0.87 (0.10) | 0.85 (0.09) | 0.89 (0.08) | 0.90 (0.08) | 0.86 (0.12) | 0.87 (0.08) |
| Fraction Correct Trials | 0.82 (0.10) | 0.82 (0.09) | 0.84 (0.11) | 0.85 (0.10) | 0.83 (0.10) | 0.82 (0.11) |
| Cumulative reward | 117 (4.45) | 117 (3.61) | 117 (2.81) | 117 (3.81) | 115 (9.27) | 114 (8.20) |


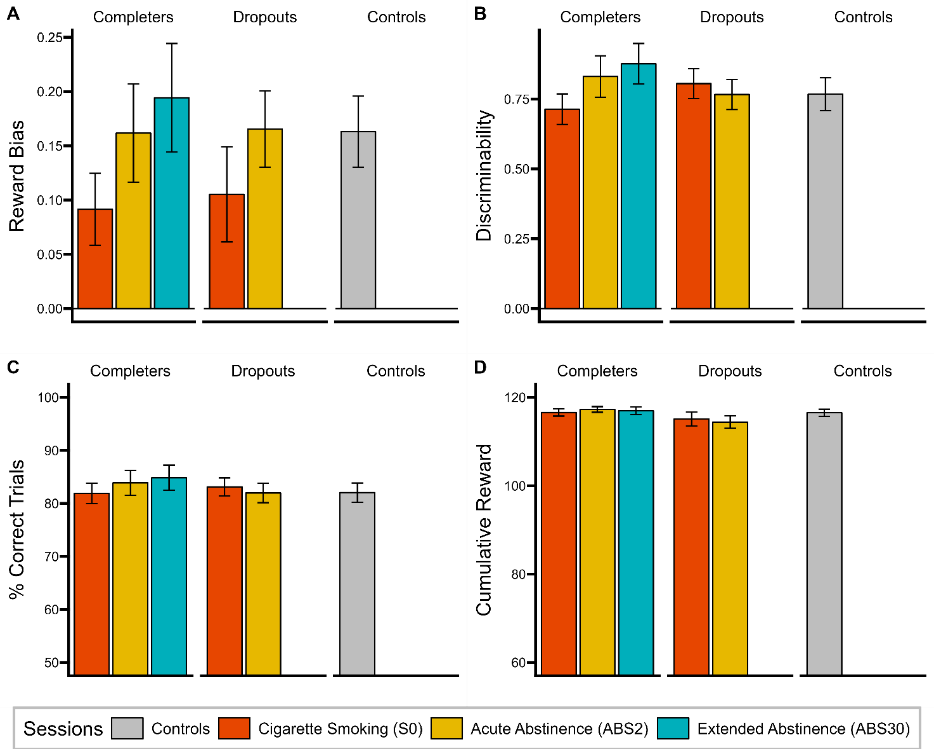


***Figure S3. Signal Detection Analyses.*** Mean reward bias (A), discriminability (B), correct responses (%, C) and cumulative rewards (D) by study group and smoking status. Error bars represent standard errors.

***Figure S4. Sensitivity to losses according to the signal detection analysis.*** Percentage of correct trials following negative trials where no rewards where delivered (A) and successful trials (B) by block, study group and smoking status. Error bars represent standard errors.

## Model-based results.

### Model fitting and model selection

The model ‘Action’ and the model ‘Punishment’ performed better than the basic Rescorla-Wagner model ‘Stimulus-action’ and showed better iBIC values compared to all the other models (**Figure S5**). The difference between the two models - ‘Punishment’ and ‘Action’ - was small (Δ iBIC = 2.1) and did not allow us to choose the best model between the two. In line with our preregistered hypotheses, these results imply that participants were not able to completely separate the two stimuli while learning (action only) and that they considered aversive the absence of rewards, modifying their behavior accordingly.


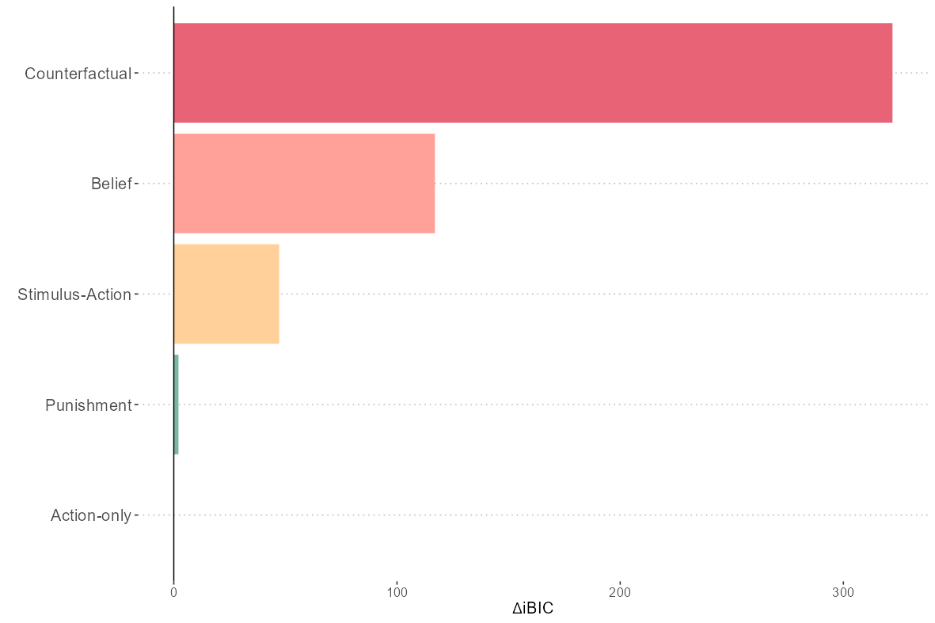
 **Figure S5. Model comparison by group level log Bayes factors**. Here reported the ∆iBIC defined as the difference between the model with the lowest iBIC and each of the remaining four models.

The model ‘Belief’ has been previously shown to better explain data from different samples, including healthy subjects and patients with mood disorders (3). However, given the specific characteristics of our sample and the specific role of negative reinforcement in nicotine addiction and withdrawal, we were particularly interested in the model ‘Punishment’. The punishment model resembles the basic stimulus-action Rescorla-Wagner model but has two separate sensitivity factors for reward and non-reward events. This model considers that individuals are perceiving non-rewards as punishments, but the assumption that learning is based on the stimulus-action pairing is maintained.

### Model-based parameters

#### ‘Punishment’ model

Punishment model-based parameters are reported in Table S3. Similarly to previously described procedures (3), to assess the recovery of model parameters, we implemented a series of simulations using the maximum a posteriori (MAP) estimation method. Parameter estimates were generated for key reinforcement learning parameters, including reward sensitivity (β), learning rate (α), initial bias (Q0), and instruction sensitivity (γ). The recovery results in **Figure S6** illustrate the distribution of MAP estimates for each parameter in the entire sample. The box plots indicate consistent recovery of parameters, with the median estimates aligning closely with expected theoretical values. Minimal variability and the absence of significant outliers suggest that the model effectively captures the underlying dynamics of the decision-making process. These findings enhance confidence in the robustness and applicability of the model in understanding behavioral responses.

Also, to evaluate multicollinearity among the model parameters, we computed the correlation coefficients and the Variance Inflation Factor (VIF) for each session. The correlation matrices revealed moderate correlations, with the highest value being 0.8 for PUN and alpha in Session 1 (S0). VIF values were all below 2.0 across sessions, with the highest measurement being 1.60 in Session 3 (ABS30). These results indicate that multicollinearity is not a concern in this analysis, suggesting that the model parameters can be reliably estimated without significant redundancy.

In **Table S3**, we provided a comprehensive summary of the key parameters analyzed in our study, highlighting their significance and variations across the dataset. Additionally, we conducted a comparative analysis of different groups (Controls, Dropouts and Completers), which is visually represented in **Figure S7**.

Also, to better understand the influence of punishment sensitivity (β_punishment_) on choice probabilities and task performance, using similarity matrices based on the Euclidean distance between participants’ parameter scores, we extracted pairs of subjects that exhibited significantly different levels of punishment sensitivity but were comparable in other parameters. Contrasting task performance (both cumulative rewards and cumulative accuracy) in each pair, we did not observe any discernible differences in trial-by-trial cumulative reward curves, while subjects with higher punishment sensitivity demonstrated greater trial-by-trial accuracy for both lean and rich trials (see **Figure S8**). This supports the notion that an increased punishment sensitivity may lead subjects to avoid missed rewards, increasing their ability to discriminate between action outcomes, allowing for more precise behavior in response to negative feedback. At the same time, higher punishment sensitivity seems to lead to greater responsiveness to suboptimal choices, resulting in adjustments that enhance accuracy without altering task performance in terms of reward.

**Table S3. ‘Punishment model’ parameters.** Mean values (standard deviations) of log-transformed parameters by study group and smoking status.

|  | Controls n: 31 | Completers N: 19 | | | DROPOUTS N: 34 | |
| --- | --- | --- | --- | --- | --- | --- |
|  | Baseline | Cigarette Smoking | Acute Abstinence | Extended Abstinence | Cigarette Smoking | Acute Abstinence |
| REWARD SENSITIVITY  log(β_reward_) | 0.19 (0.87) | 0.12 (0.78) | 0.16 (0.69) | 0.14 (0.59) | 0.03 (0.75) | 0.09 (0.79) |
| PUNISHMENT SENSITIVITY  log(β_punishment_) | 0.66 (1.58) | -0.10 (1.93) | 0.56 (1.56) | 1.53 (0.94) | 0.27 (1.80) | 0.22 (1.89) |
| INSTRUCTION SENSITIVITY  log(γ) | 0.57 (0.55) | 0.42 (0.43) | 0.68 (0.53) | 0.96 (0.37) | 0.43 (0.58) | 0.36 (0.67) |
| LEARNING RATE  log(α/(1-α)) | -3.64 (1.97) | -2.66 (2.39) | -3.10 (1.68) | -3.94 (1.58) | -3.49 (2.25) | -3.63 (2.36) |
| INITIAL BIAS  log(Q_0_) | -0.18 (0.60) | 0.04 (0.62) | -0.14 (0.67) | 0.34 (0.68) | -0.18 (0.78) | -0.15 (0.72) |


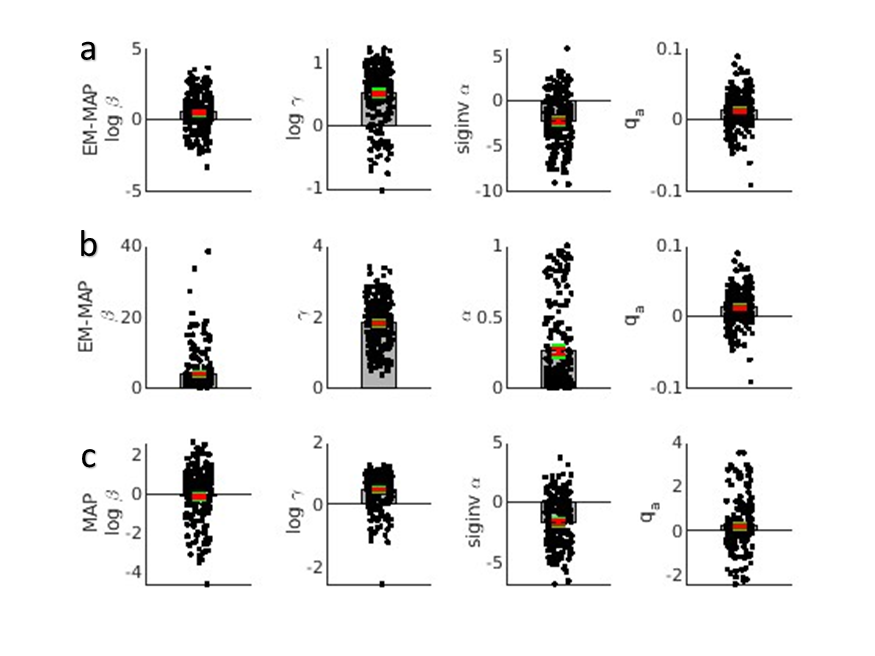


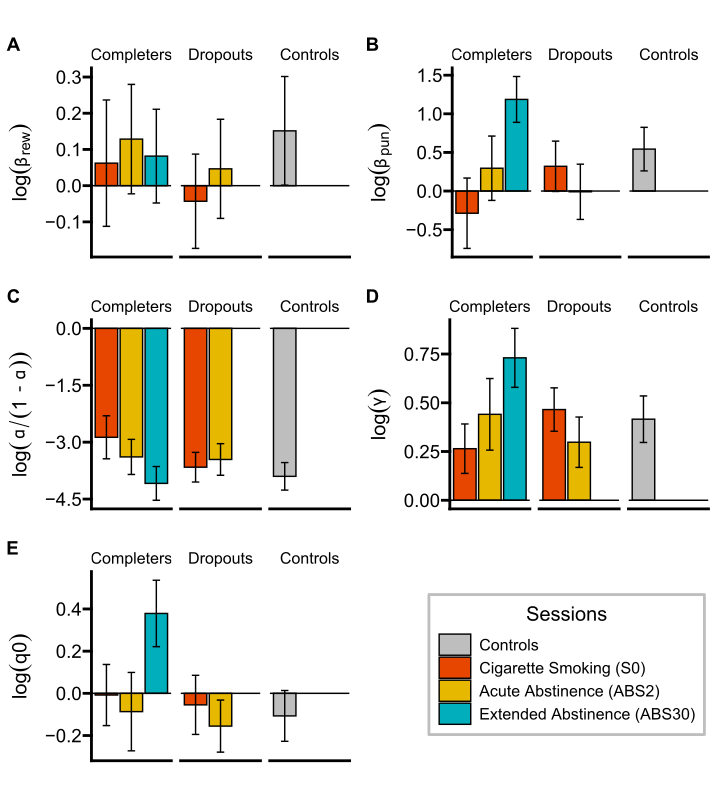


**Figure S6. Parameter recovery analysis**. Distribution of Expectation-Maximization Maximum A Posteriori (EM-MAP) estimates for key transformed (a) and non-transformed (b) reinforcement learning parameters, and the distribution of Maximum A Posteriori (MAP) for transformed parameters. Reported parameters: reward sensitivity (β), learning rate (α), initial bias (q0), and instruction sensitivity (γ).

***Figure S7.*** ***‘Punishment’ model output.*** Mean reward sensitivity (log(β_reward_), A), punishment sensitivity (log(β_punishment_), B), learning rate (log(α/(1-α)), C), and instruction sensitivity (log(γ), D) by study group and smoking status. Error bars represent standard errors.


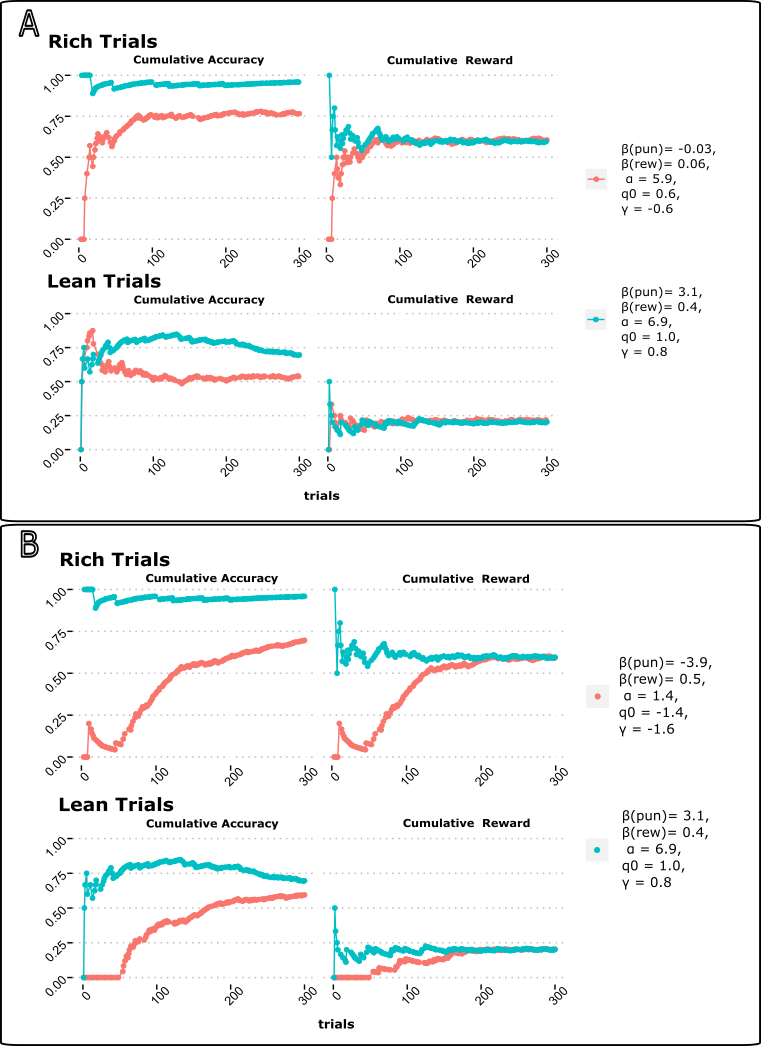


**Figure S8.** **Effects of varying reward and punishment sensitivity parameters on model performance in rich and lean trial conditions.** Two pairs of subjects with distinct parameter sets are shown (A and B), each contrasting task performance for a subject with high-punishment sensitivity (blue) with a subject with low-punishment sensitivity (orange).

#### ‘Action-only’ model

**Table S4. ‘Action-only model’ parameters.** Mean values (standard deviations) of log-transformed parameters by study group and smoking status.

|  | Controls n: 31 | Completers N: 19 | | | DROPOUTS N: 34 | |
| --- | --- | --- | --- | --- | --- | --- |
|  | Baseline | Cigarette Smoking | Acute Abstinence | Extended Abstinence | Cigarette Smoking | Acute Abstinence |
| REWARD SENSITIVITY  log(β_reward_) | 0.80 (1.30) | 0.47 (1.62) | 0.63 (1.21) | 0.69 (1.30) | 0.33 (1.59) | 0.48 (1.37) |
| INSTRUCTION SENSITIVITY  log(γ) | -2.72 (2.71) | 0.45 (0.38) | 0.58 (0.45) | 0.62 (0.46) | -1.71 (3.20) | -2.00 (2.82) |
| LEARNING RATE  log(α/(1-α)) | 0.49 (0.44) | -2.09 (3.59) | -2.38 (2.45) | -2.60 (2.50) | 0.52 (0.45) | 0.46 (0.49) |
| INITIAL BIAS  log(Q_0_) | 0.01 (0.03) | 0.02 (0.02) | 0.01 (0.03) | 0.02 (0.02) | 0.02 (0.03) | 0.02 (0.02) |

## Association between subjective measures of craving and withdrawal, ‘Punishment’ parameters and model-free variables.

To better understand the meaning of the ‘Punishment’ model’s parameters and further validate it, we looked at the association between each model’s parameter and the model-free variables. As expected and has noted previously (3), we found a significant and positive association between the information integration parameter (γ) and the accuracy expressed as fraction of correct responses (FCR) (r=0.849; p<.0.001) (**Figure S9**).


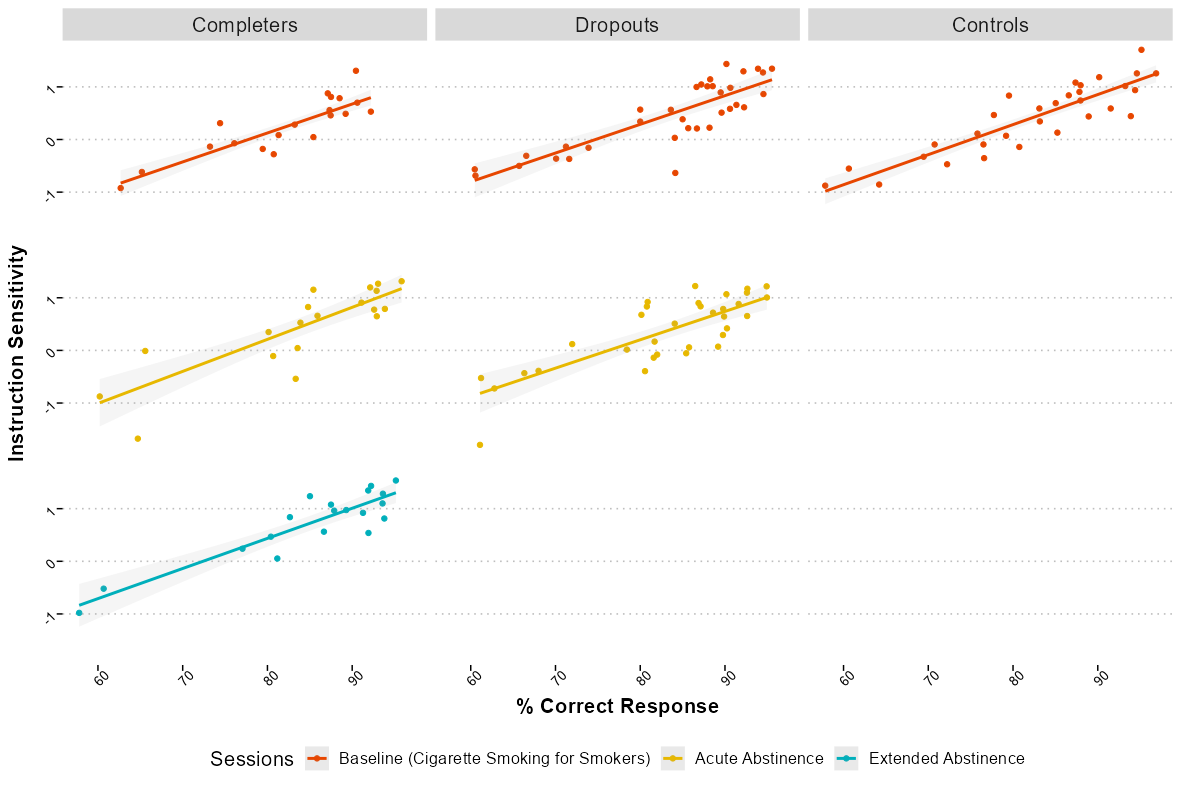


**Figure S9. Relationship between instruction sensitivity (log(**γ**)) and percentage of correct responses.** Correlation plots and lines by study group and smoking status.

Also, a strong negative correlation between learning rate and punishment sensitivity (r = -0.76, p < .001) was observed in the entire sample, while there was only a weak relationship between learning rate and reward sensitivity (r = -0.19, p = .017) (**Figure S10**). The latter did not survive when we separately analyzed the group of smokers who completed the smoking cessation protocol (**Figure S11**) and dropouts (**Figure S12**).

Notably, when analyzed separately, the relationship between learning rate and punishment sensitivity was stronger in sated smokers – both completers (r = -0.85, p < .001) and dropouts (r = -0.81, p < .001) – than in controls (r = -0.65, p < .05), suggesting smokers have a higher propensity to modulate behavior as a function of negative prior reinforcement compared to non-smokers comparisons. Furthermore, while the correlation between ε and -ρ remained stable at ABS2 in both completers (r = -0.7, p < .001) and dropouts (r = -0.81, p < .001), it was weaker at ABS30 (r = -0.5, p < .05), suggesting a possible switch in learning strategies during prolonged abstinence.


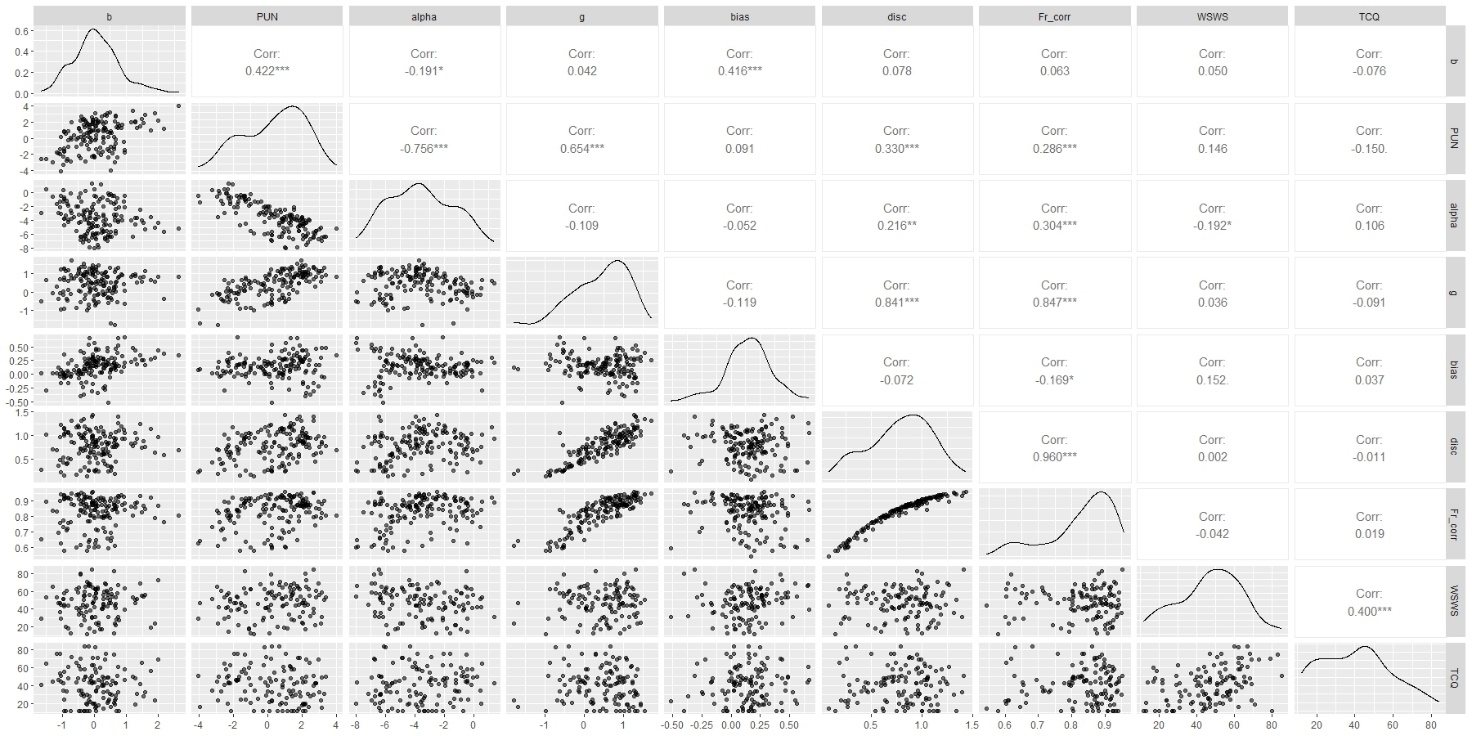


**Figure S10.** **Correlations between punishment model parameters, model-free variables and subjective measures of withdrawal in the entire sample (n = 84).** Correlation plots and coefficients for all pairwise correlations between model-based parameters (reward sensitivity (b), punishment sensitivity (PUN), instruction sensitivity (g), learning rate (alpha)) and model-free variables (reward bias (bias), discriminability (disc), and percentage of correct responses (Fr_corr)), and craving (TCQ) and withdrawal symptoms (WSWS) in the entire sample, including all timepoints.*: p < 0.5, **: p < 0.01, ***: p < 0.001.


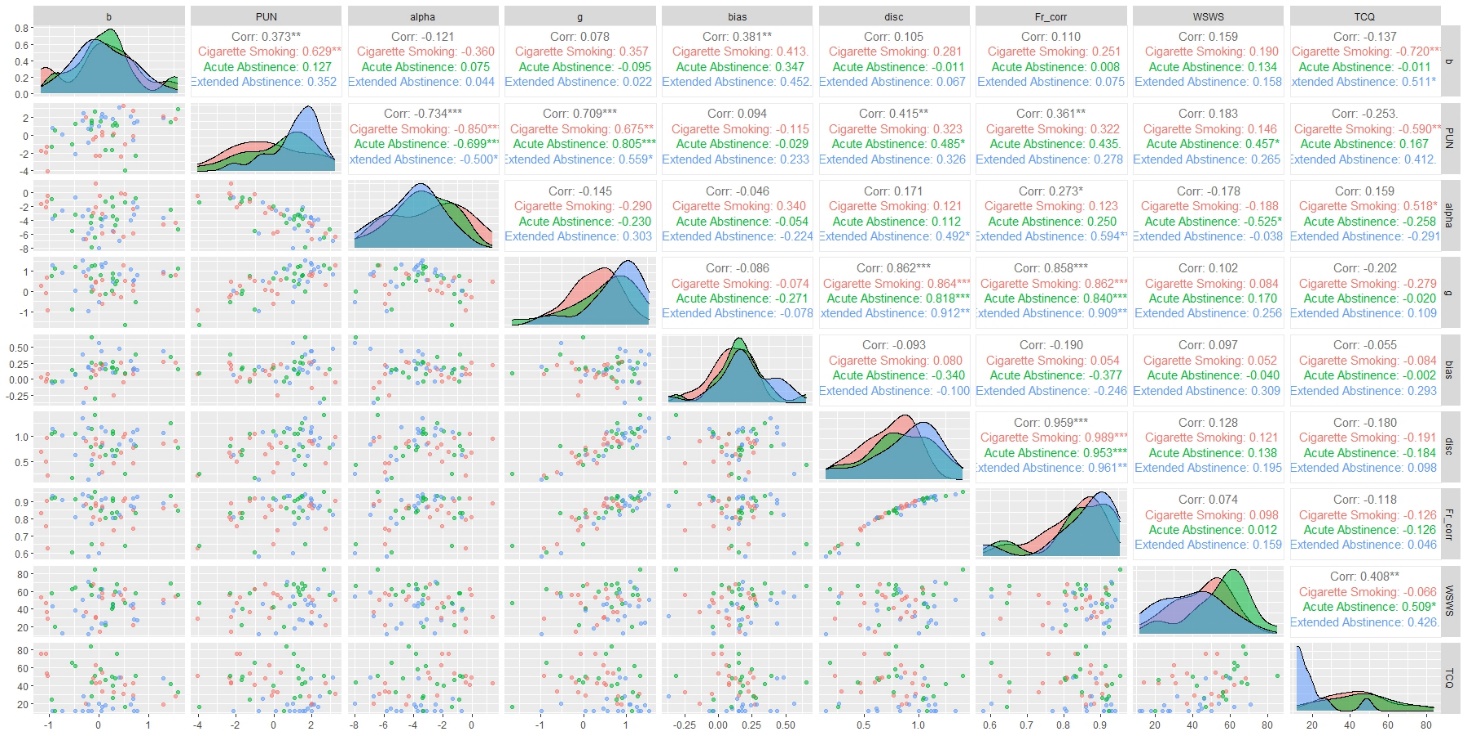


**Figure S11. Correlations between punishment model parameters, model-free variables and subjective measures of withdrawal in completers.** Correlation plots and coefficients for all pairwise correlations between model-based parameters (reward sensitivity (b), punishment sensitivity (PUN), instruction sensitivity (g), learning rate (alpha)) and model-free variables (reward bias (bias), discriminability (disc), and percentage of correct responses (Fr_corr)), and craving (TCQ) and withdrawal symptoms (WSWS). Correlation coefficients and distributions are calculated separately for each timepoint (Baseline (in red), Acute Abstinence (in green) and Extended Abstinence (in blue)). *: p < 0.5, **: p < 0.01, ***: p < 0.001.


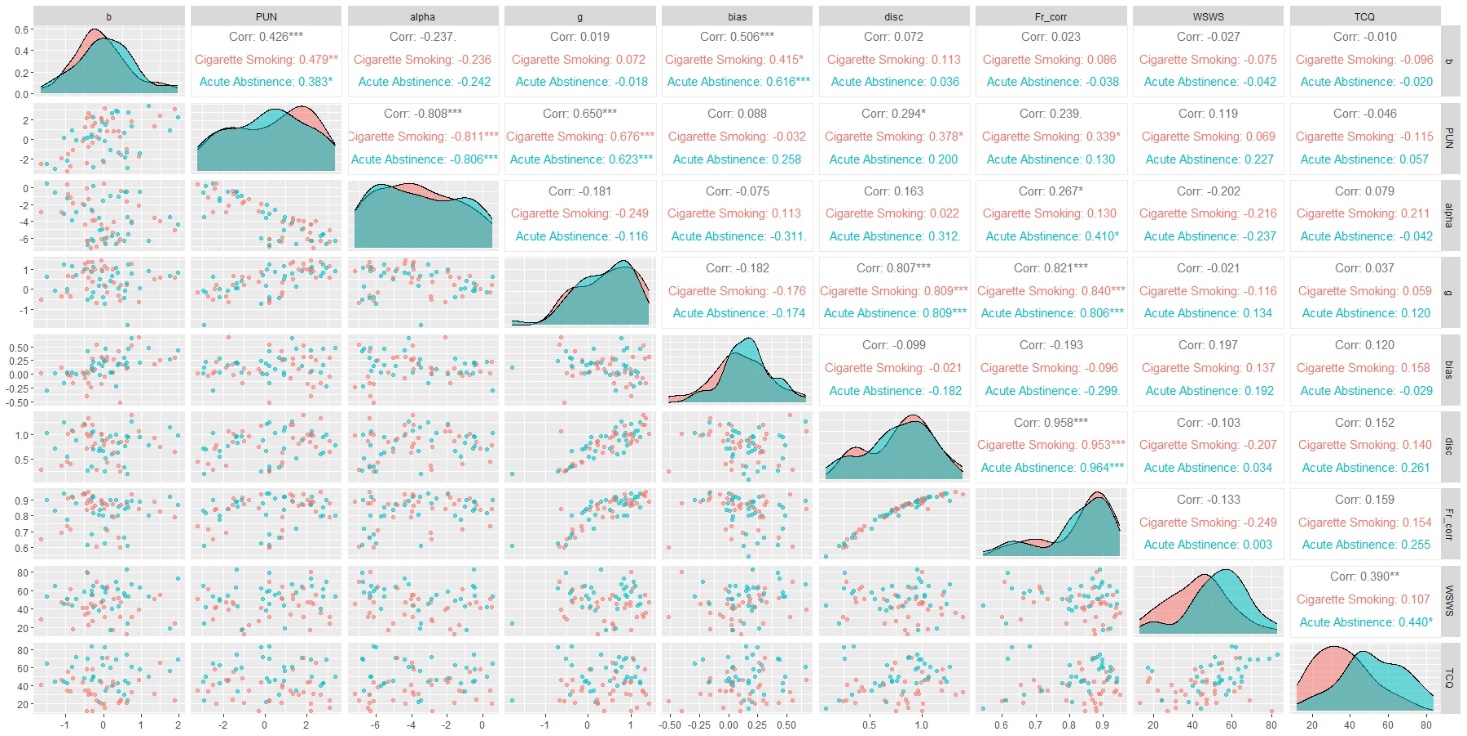


**Figure S12. Correlations between punishment model parameters, model-free variables and subjective measures of withdrawal in dropouts.** Correlation plots and coefficients for all pairwise correlations between model-based parameters (reward sensitivity (b), punishment sensitivity (PUN), instruction sensitivity (g), learning rate (alpha)) and model-free variables (reward bias (bias), discriminability (disc), and percentage of correct responses (Fr_corr)), and craving (TCQ) and withdrawal symptoms (WSWS). Correlation coefficients and distributions are calculated separately for each timepoint (Baseline (in red), Acute Abstinence (in green)). *: p < 0.5, **: p < 0.01, ***: p < 0.001.

## Association between subjective measures of craving and withdrawal, ‘Action-only’ parameters and model-free variables.

Similarly, we analyzed the association between the Action-only model parameters and model-free variables and subjective measures of withdrawal, in completers (**Figure S13**). We did not find any association between the model-free variables and the subjective measures of craving and withdrawal in completers. Moreover, we did not observe any repeated-measure, within-subject correlations between Punishment model’s parameters and withdrawal syndrome (WSWS) in completers (β_punishment_: r=-0.16, p=0.3; β_reward_: r=-0.03, p=0.8; α: r=-0.13, p=0.4). Notably, when separately evaluated at each timepoint, a significant negative correlation was observed between WSWS and both punishment sensitivity and learning rate, but not reward sensitivity, in completers only at ABS2 (β_punishment_: r=0.46, p=0.05; β_reward_: r=0.13, p=0.58; α: r=52, p=0.02) (**see Figure S11, S13**).

Also, nor model-based parameters or model-free variables show any significant association with subjective measures of craving and withdrawal in dropouts.


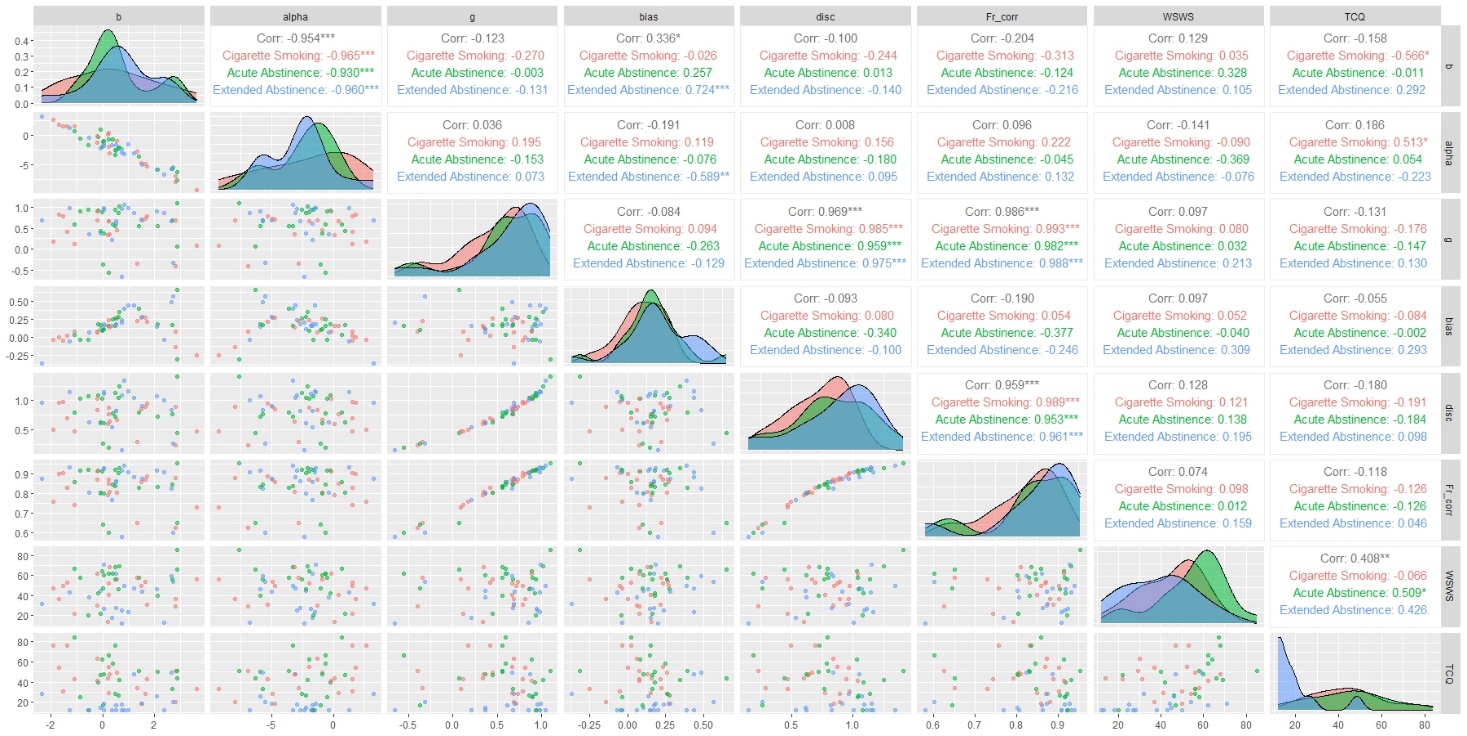
 **Figure S13.** **Correlations between action-only model parameters, model-free variables and subjective measures of withdrawal in completers.** Correlation plots and coefficients for all pairwise correlations between model-based parameters (reward sensitivity (b), punishment sensitivity (PUN), instruction sensitivity (g), learning rate (alpha)) and model-free variables (reward bias (bias), discriminability (disc), and percentage of correct responses (Fr_corr)), and craving (TCQ) and withdrawal symptoms (WSWS). Correlation coefficients and distributions are calculated separately for each timepoint (Baseline (in red), Acute Abstinence (in green) and Extended Abstinence (in blue)). *: p < 0.5, **: p < 0.01, ***: p < 0.001.

# References

1. Macmillan NA, Creelman CD. Detection theory: A user's guide, 2nd ed. Mahwah, NJ, US: Lawrence Erlbaum Associates Publishers; 2005. xix, 492-xix, p.

2. Pizzagalli DA, Jahn AL, O’Shea JP. Toward an objective characterization of an anhedonic phenotype: A signal-detection approach. Biological Psychiatry. 2005;57(4):319-27.

3. Huys QJM, Pizzagalli DA, Bogdan R, Dayan P. Mapping anhedonia onto reinforcement learning: a behavioural meta-analysis. Biology of Mood & Anxiety Disorders. 2013;3(1):12.

4. Raftery AE. Bayesian Model Selection in Social Research. Sociological Methodology. 1995;25:111-63.

5. Kass R, Raftery A. Bayes Factor. Journal of American Statistical Association. 1995;90(430):773-95.

6. Pergadia ML, Der-Avakian A, D’Souza MS, Madden PAF, Heath AC, Shiffman S, et al. Association Between Nicotine Withdrawal and Reward Responsiveness in Humans and Rats. JAMA Psychiatry. 2014;71(11):1238-45.
